# The samplr package: A tool for modeling human cognition with sampling algorithms

**DOI:** 10.3758/s13428-026-03188-6

**Published:** 2026-10-14

**Authors:** Lucas Castillo, Yun-Xiao Li, Adam N. Sanborn

**Affiliations:** University of Warwick, Coventry, UK

**Keywords:** Sampling, R package, MCMC, Bayesian

## Abstract

Many models of cognition have sampling as a central component. Researchers have used them to explain human performance across a wide range of tasks involving perception, probability reasoning, and choice. One family of these models takes inspiration from sampling approaches developed in statistics, either by adjusting samples with prior beliefs or by implementing a sampling algorithm that overcomes the difficulty of independent samples at the expense of autocorrelation. While these models have been successful in modeling human behavior, their adoption has been slowed by the technical difficulties in applying them. Here, we introduce the samplr package, which includes a variety of these algorithms and cognitive models built upon them. We show how models in this package can match qualitative patterns found in human data, and walk readers through examples of how to compare these models quantitatively to human data. This tool aims to make these methods accessible to many more researchers.

## Introduction

Computational models are precise formalizations of theories that organize and condense research findings and can make predictions for novel experimental designs. In studies of cognition, many computational models rely on the computational motif of *sampling*. For example, sequential sampling models (e.g., the drift diffusion model, linear ballistic accumulator, and exemplar-based random walk) assume that sampling of percepts or memories drives an internal accumulation process that maps to human choices and response times (Brown & Heathcote, 2008; Nosofsky & Palmeri, 1997; Ratcliff & McKoon, 2008). These sequential sampling models have been augmented to explain not only choices but also metacognitive judgments such as choice confidence (e.g., Hellmann, Zehetleitner, & Rausch, 2023; Pleskac & Busemeyer, 2010).

Other kinds of sampling models have been used to explain continuous estimates (Smith, 2016) and there has been work to integrate choices, estimates, and response times (Kvam, Marley, & Heathcote, 2023). Separate models have used sampling to explain human-generated confidence intervals (Juslin, Winman, & Hansson, 2007). Finally, another class of sampling models has been used to explain people’s probability judgments, one that assumes the fallacies people show in this task are caused by inaccurate sample counting (Costello & Watts, 2014).

Computer scientists and statisticians also use sampling algorithms in their work, but for a practical purpose: to efficiently approximate complex probability distributions that otherwise would be intractable. These sampling algorithms use various tricks to provide efficient estimates. One trick, Bayesian Monte Carlo, improves the accuracy of estimates through the use of prior information (Rasmussen & Ghahramani, 2003). For example, when estimating the probability of an event through sampling, a prior distribution for the estimates is selected, and this prior distribution is updated by the samples generated by the sampling algorithm. This process improves the estimate based on a finite number of samples – in the extreme case of a single sample it moderates the resulting estimate away from 0 or 1 – and so can save time that otherwise would have been spent generating a larger number of samples. This trick improves efficiency, but as a consequence it can cause probability estimates to disagree with one another, producing the same probabilistic reasoning fallacies that people show in this task (Zhu, Sanborn, & Chater, 2020).

A second and more commonly used trick for improving the efficiency of sampling is to change the nature of the algorithm itself. Sampling directly and independently from the probabilities, as most cognitive models of sampling assume, requires knowing the probability of each and every event. This is feasible when the form of the distribution is known (e.g., Gaussian) or the number of events is small, but outside of these restricted situations it is much more difficult. For example, in a belief updating task, one may know the prior probability and the likelihood of an event, and the first step of calculating the posterior distribution of interest is to multiply the prior and likelihood together. This is easy, but the next step, dividing by the normalizing constant (i.e., the sum of each prior and likelihood across all possible events), which is needed to ensure that the distribution sums to one, can be intractably difficult. Computer scientists and statisticians have thus developed a family of algorithms, Markov chain Monte Carlo (MCMC; Metropolis, Rosenbluth, Rosenbluth, Teller, & Teller, 1953; Neal, 1993; Van Ravenzwaaij, Cassey, & Brown, 2018), that avoid the need to calculate the normalizing constant by only requiring ratios of probabilities to run – here the normalizing constants will cancel, and are not needed. The cost of these algorithms, however, is that they introduce autocorrelation, making the sample collection less informative than an independent set of samples would be, and introducing dependence on the starting point. Such dependencies are like those found in repeated judgments of people (Zhu, León-Villagrá, Chater, & Sanborn, 2022) and in the anchoring effect (Lieder, Griffiths, & Hsu, 2018).

Because statistical sampling algorithms were developed as general-purpose, efficient approximations, and because they align with the sampling motif found in models of human behavior, they are interesting candidates for modeling this behavior. However, although this approach was proposed more than two decades ago (Hoyer & Hyvärinen, 2002) and has been successfully applied across a range of tasks, it appears only intermittently in the literature.

We believe one of the reasons for such sparseness is the difficulty in applying sampling algorithms to explain human behavior. Therefore, taking inspiration from the success of software packages implementing sequential sampling algorithms (Singmann et al., 2018; Vandekerckhove & Tuerlinckx, 2008; Wiecki, Sofer, & Frank, 2013), here we offer an R package, available in the CRAN repository, that implements the different statistical sampling algorithms. Our goals are to increase the coherence of separate investigations and to increase their efficiency. We accomplish both of these goals by providing a code base, the samplr package, in which many of the functions are implemented in !C++! for speed.

**Fig. 1:**
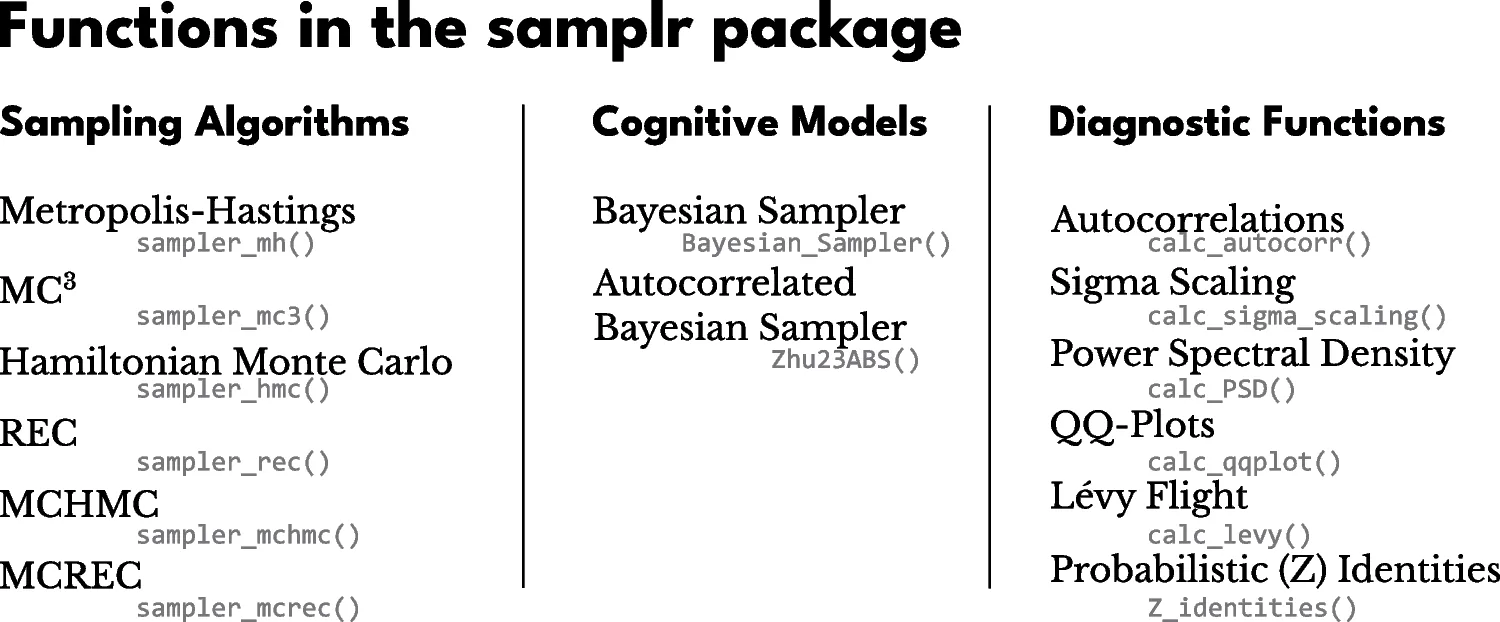
Inventory of functions currently implemented in the samplr package. In addition to several statistical sampling algorithms whose behavior has previously been compared to human data, the package offers cognitive models, as well as a range of diagnostic functions which can be used when examining model performance quantitative or qualitatively. In *grey*, names of the functions in the package

The samplr package (see Fig. 1) includes a variety of MCMC sampling algorithms – such as Metropolis–Hastings (MH), Hamiltonian Monte Carlo (HMC), and their variants – as well as cognitive models like the Bayesian sampler (Zhu et al., 2020) and the autocorrelated Bayesian sampler (ABS; Zhu, Sundh, Spicer, Chater, & Sanborn 2024). Because of their implementation in a low-level programming language, these run up to four times faster than they would if coded in MATLAB, and much faster than if coded in base R (see package vignette). The package also offers diagnostic functions for analyzing autocorrelation, power spectral density, and other features that have been used to compare sampling algorithms to human behavior, which can be used in conjunction with more domain-specific diagnostic tools experimenters might employ.

In the following sections, we first demonstrate how to use the sampling algorithms and cognitive models in the samplr package to reproduce qualitative patterns observed in empirical studies. We then show how the samplr package can be integrated with likelihood-free inference tools to enable quantitative model comparisons at the level of individual participants (in one of the tools we show, we use the external abcrf package). Throughout, we use version 1.1.2 of the package and provide short code examples with example outputs. Interested readers may also want to examine the package documentation (https://lucas-castillo.github.io/samplr/), the supplementary materials to this paper in which we show how our figures were produced (https://github.com/lucas-castillo/samplr-paper), and the Appendix, which contains implementation details.

## Qualitative patterns

First, we review examples of sampling algorithms and cognitive models qualitatively matching patterns of human behavior, across response modes such as probability judgments, estimates, choices, response times, confidence intervals and confidence reports. We also present an example of sampling algorithms explaining how response modes can interact: how choices can bias subsequent estimates, sometimes attracting and sometimes repulsing them.

### Probability judgments

A large body of research has shown that people commit systematic errors when reasoning with probabilities. For example, their probability judgments are lower if the question includes unlikely examples, as opposed to likely examples (*implicit subadditivity effect*; Dasgupta, Schulz, & Gershman, 2017). Sometimes, their judgments even violate Kolmogorov probability axioms: estimates of the probability of an event “A” may be smaller than those of the conjunction “A and B” (*conjunction fallacy*; Hertwig, Benz, & Krauss, 2008; Tversky & Kahneman, 1983); and estimates of “A” and of the converse and mutually exhaustive “not A” might add up to more than one (violation of *binary complementarity*; Huang, Busemeyer, Ebelt, & Pothos, 2024; Macchi, Osherson, & Krantz, 1999; Tee, Sundh, Chater, & Sanborn, 2024).

A useful approach to study probability judgments has been to query participants on the probabilities of two events “A” and “B”, their complements “not A” and “not B”, and their conjunctions and disjunctions (e.g., “A and not B”). These responses can then be added or subtracted to produce a set of identities (called Z identities; for example, the sum of the estimates of “A” and of “B” should equal the sum of “A and B” and “A or B”), which helps characterize systematic errors in probability reasoning (Costello & Watts, 2014, 2016, 2018).

**Fig. 2:**
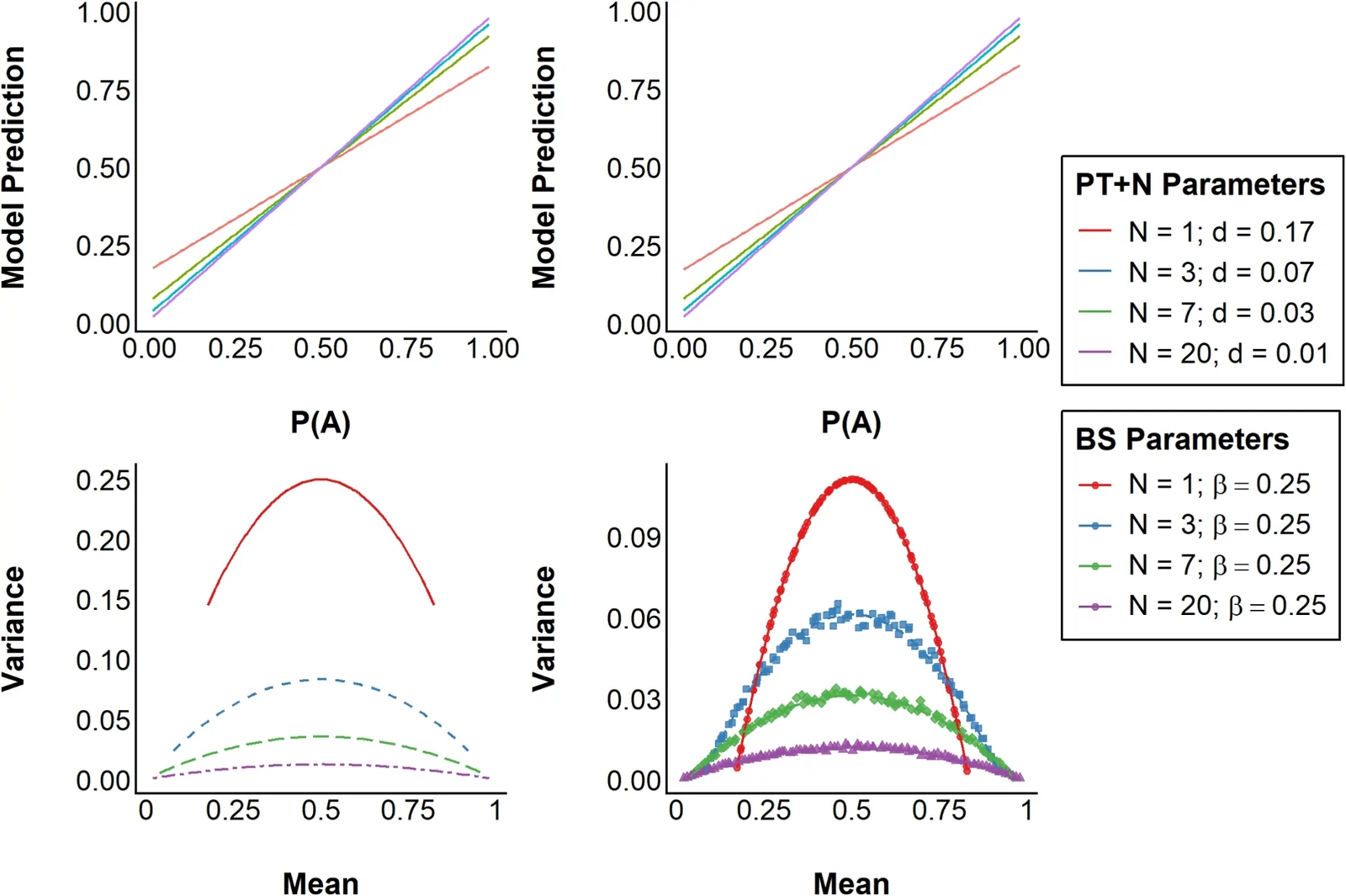
Results of the Bayesian sampler (Zhu et al., 2020) and probability theory plus noise (Costello & Watts, 2014). While model predictions are identical for mean estimates, they make different predictions regarding the mean-variance relationship (Sundh et al., 2023)

A model that explains people’s errors in probability judgments is probability theory plus noise (PT+N; Costello & Watts, 2014, 2017). According to this model, to judge the probability of an event, people sample instances of events from memory according to their true probability, but sometimes misread them (e.g., mistakenly count a sample “A” as “not A”) with a fixed probability *d*. Alternatively, according to the Bayesian sampler model, samples are never misread, but instead are adjusted by a beta prior on responses. Surprisingly, these two models make identical predictions with regards to the mean of probability judgments (Costello & Watts, 2019; Zhu et al., 2020). However, their predictions on the relationship between the mean judgments and their variance are different, due to PT+N adjusting samples as they are drawn, while the Bayesian sampler adjusts the estimate after the tally (Sundh, Zhu, Chater, & Sanborn, 2023). In the Bayesian sampler, a smaller number of samples will lead to the endpoints of the mean-variance curve approaching the center, while in PT+N the endpoints of the curve will not be affected (see Fig. 2).

With the samplr package, users can compute this set of identities by using the Z_identities function (we provide abridged and rounded example outputs when relevant, preceded by “#>”), completing parameters with the person’s estimates of the probabilities of events a and b, as well as their conjunctions (e.g., a_and_b), disjunctions (e.g., a_or_b), and conditionals (e.g., a_given_b).

**Figure:**
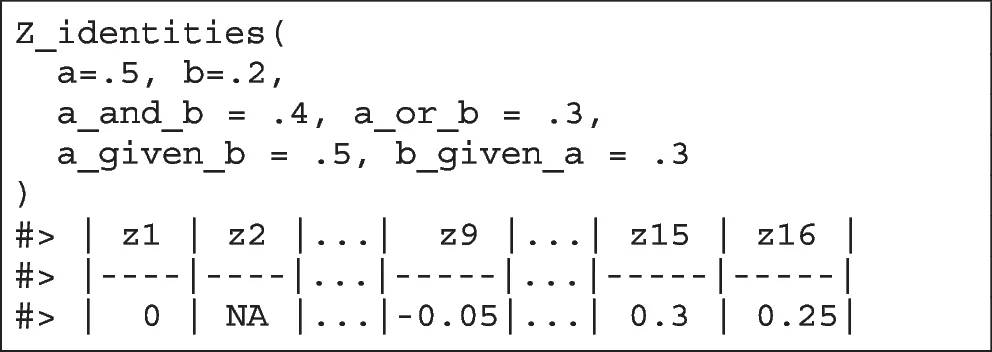

Researchers can also use the Bayesian_Sampler function to simulate and make predictions with the Bayesian sampler model; by inputting the true probabilities, the symmetric Beta prior (beta) and fixed sample size (N) parameters of the model, and whether the function should return the expected mean, expected variance, or a simulation of the outcomes:

**Figure:**
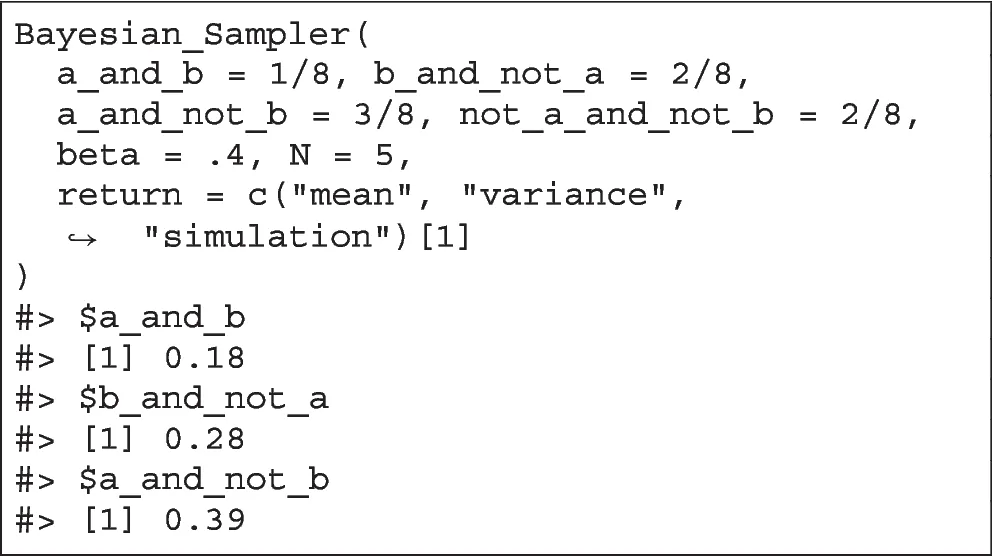

### Estimates

A surprising fact of human cognition is that people’s responses on seemingly unrelated trials can actually be predictive of one another. In Gilden, Thornton, and Mallon (1995), participants listened to an auditory stimulus and then were asked to repeatedly reproduce it, matching its duration. They found that the errors participants made were not independent of each other, but instead depended on previous errors, both recent and old. Expressed as a power law relationship ($S(f) \sim 1/f^\alpha$) between the spectral power (*S*(*f*)) and the frequency (*f*), they found that the best-fitting exponent lay between white noise (α=0; independent errors) and Brownian noise (α=2; errors dependent on previous estimate only). This in-between region, called pink or 1/*f* noise ($\alpha \in [0.5, 1.5]$), represents long-range autocorrelations between estimates (Gardner, 1978).

Within a sampling framework, these temporal estimates can be characterized as samples drawn without aggregation into an inferential decision. Zhu, León-Villagrá, Chater, & Sanborn (2022) replicated Gilden et al. (1995) result, and used the resulting sequences to compare different sampling algorithms that might show the same structure in estimate errors. They compared three different sampling algorithms, Metropolis–Hastings (MH; Hastings, 1970), Metropolis-coupled MCMC (MC^3^; Geyer, 1991) and Hamiltonian Monte Carlo (HMC;  Neal, 2011), in their ability to produce long-range autocorrelations in their estimates. Unlike the samples produced by the Bayesian sampler above, these algorithms produce autocorrelated samples. Intuitively, MH iteratively produces a chain of samples by adding Brownian noise to the previous estimate, with an accept/reject stage that probabilistically discards a proposed sample if its density in the target distribution is smaller than the previous estimate.

**Fig. 3:**
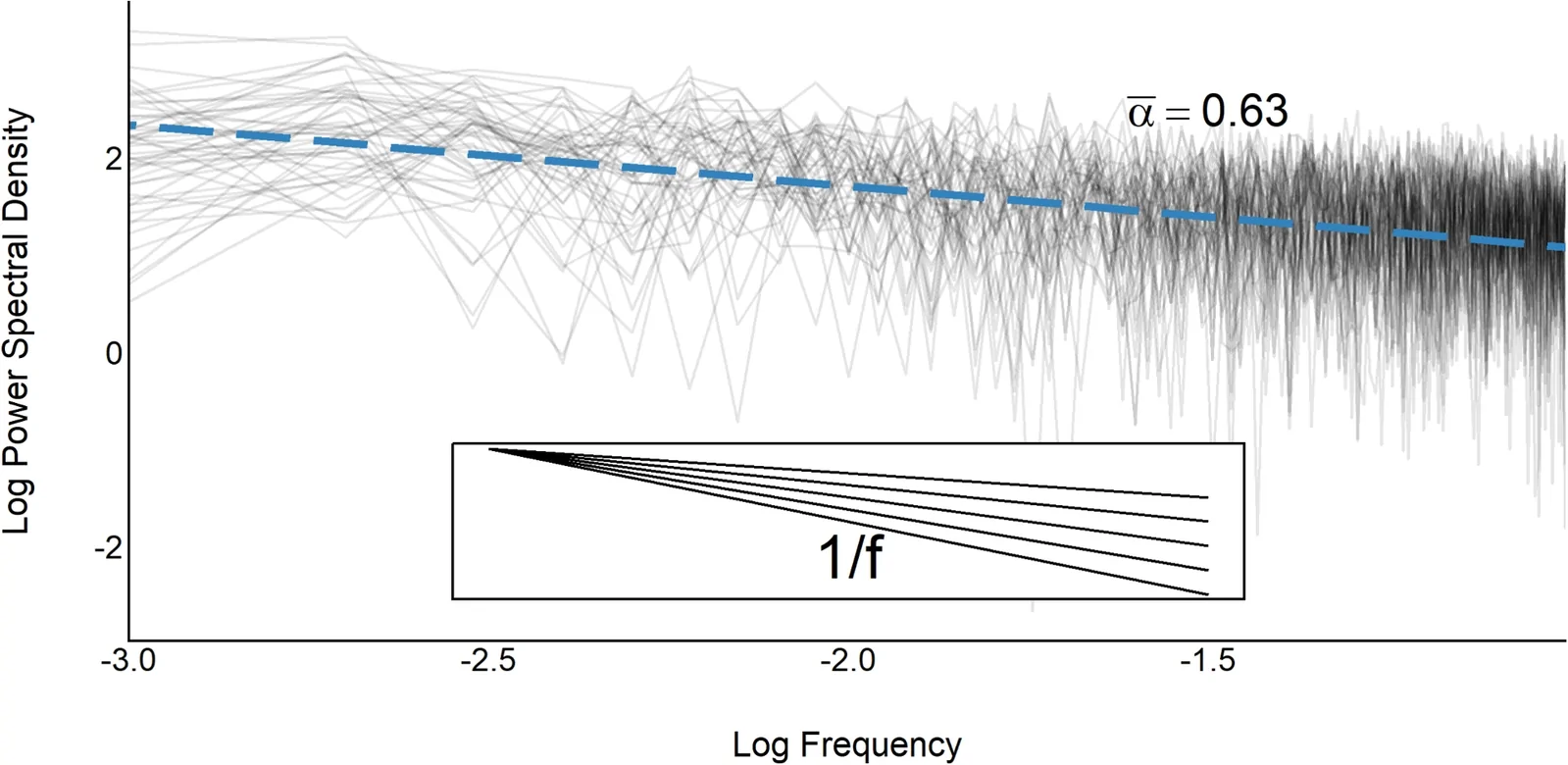
Power spectral analysis of 50 simulations of MC^3^, estimating a normal distribution. The relationship between the spectral power *S*(*f*) and the frequency *f* is best described as a power law $S(f) \sim 1/f^\alpha$ with exponents $\alpha \in [0.5, 1.5]$, consistent with 1/*f* noise (for convenience we show this range in the inset)

MC^3^ and HMC modify MH in different ways: MC^3^ runs several chains which occasionally swap positions, allowing for a chain to show large differences in consecutive estimates after a swap. HMC proposes new items in a more sophisticated way than adding noise (by simulating a physical system on the target distribution), and is thus less likely to reject proposals. Zhu et al. found that the algorithm running multiple chains (MC^3^) performed best and was able to reproduce the long-range autocorrelations found in human estimates (see Fig. 3).

Another task where sampling algorithms have been used to understand human performance is the random generation task (Castillo, León-Villagrà, Chater, & Sanborn, 2024a; Li et al., 2024). In this task, participants are given a domain of interest (e.g., numbers one to ten; human heights) and they have to produce a sequence of items from this domain as if drawn “out of a hat” with replacement, i.e. completely independent of each other. A large body of research has shown that people’s sequences deviate from true random ones in systematic ways (Baddeley, 1966; Towse, 1998; Wagenaar, 1972). For example, the distance between items in people’s sequences is too small, and people almost never repeat the same item twice in a row.

In two experiments, (Castillo et al., 2024a) compared six sampling algorithms and a previous random generation model (Cooper, 2016) against human performance. Participants produced random heights, which in one condition were distributed as in the general population (*Gaussian* condition) whilst in the other they were all equally likely (*Uniform* condition).

**Fig. 4:**
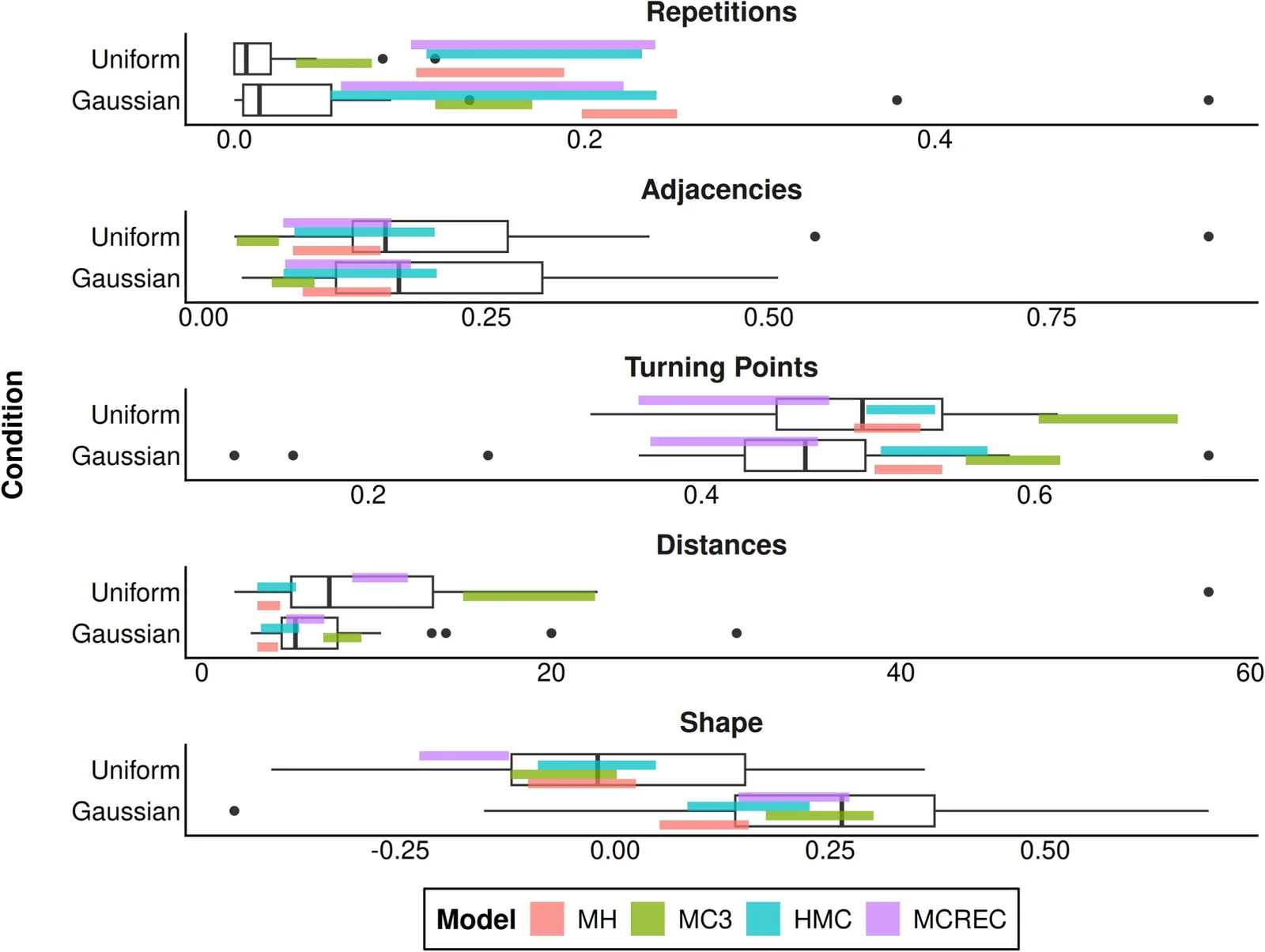
Random generation patterns (see Table 1). Boxplots show data from Experiment 1 in Castillo et al. (2024a), where participants produced random heights, for the uniform (all heights equally likely) and Gaussian (heights distributed as in the general population) conditions. *Rectangles* show the mean values of simulations ± 0.5 standard deviations, using parameters drawn from their prior, for MCREC (the model that would best fit the data) and three popular MCMC algorithms

The authors focused on the models’ ability to replicate four patterns of sequential dependence found in human data as well as people’s ability to match non-uniform distributions (see Table 1). They found that the best-fitting model was MCREC, a sampling algorithm combining the proposal mechanism of HMC with the multiple chains idea of MC^3^ and with a sense of momentum, which adds autocorrelation to the direction of proposals (we compare data to prior predictives of these models in Fig. 4).

**Table 1:** Random generation measures. *Note:* ⇊ too low ⇈ too high ✓ correct

Measure	Description	Human Behaviour
Repetitions	% items equal to last	⇊
Adjacencies	% items one unit away from last	⇈
Turning Points	% of trajectory changes (asc. to desc. or vice versa)	⇊
Distances	Mean distance between consecutive items	⇊
Shape	Relative fit of Gaussian vs. Uniform densities	✓

These six MCMC sampling algorithms are available in the samplr package, in functions starting with sampler_*. Users must indicate the target distribution and a starting point, as well as sampler-specific parameters. For example, the following code generates samples from a standard normal distribution using the classic Metropolis–Hastings algorithm:

**Figure:**
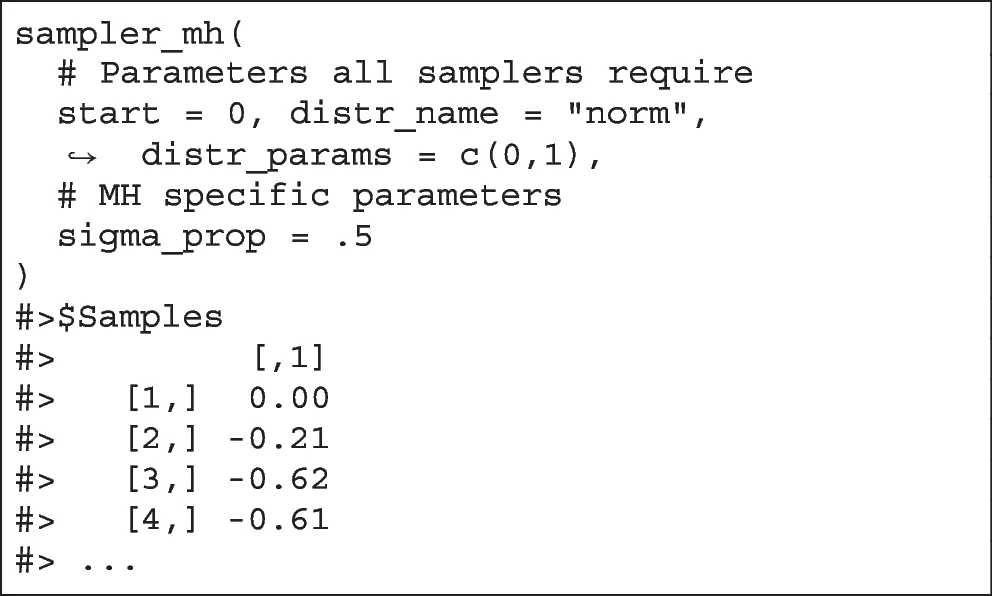

These functions can sample from mixture distributions

**Figure:**
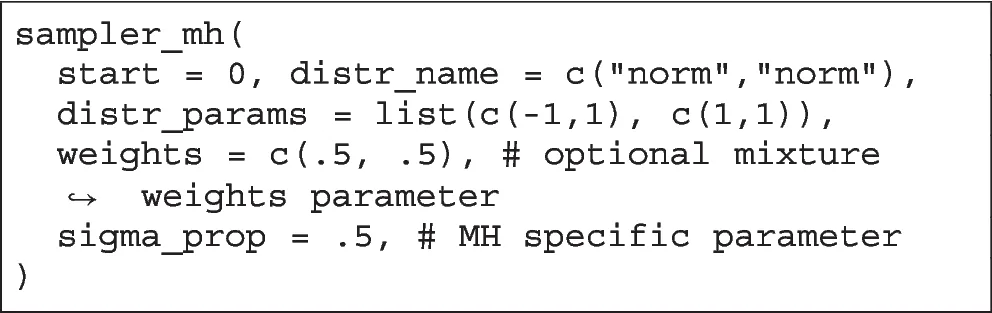

as well as bespoke distributions:

**Figure:**
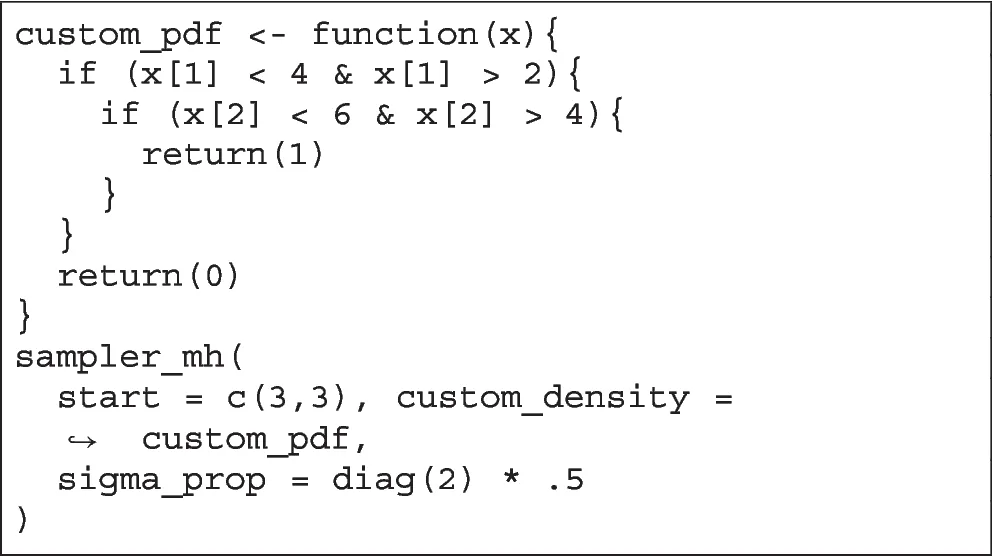

#### Case study – sequentially estimating a latent variable

In this section, we use research by Bruckner, Nassar, Li, and Eppinger (2025) to show how the sampling algorithms available in the samplr package have been used in modeling cognition by other research groups. We use this case study to demonstrate how these modeling tasks can be approached using the samplr package for increased ease.

Bruckner et al. (2025) were interested in studying how people navigate situations in which the variability in sequential data might be due to typical fluctuations from a constant latent state, or to a change in the latent state altogether (e.g., a subpar meal at an often-visited restaurant being due to transitory bad luck or a permanent change of cook). In learning tasks where people repeatedly estimate the true latent mean of the generating process from data, people exhibit two prominent biases: perseveration, so that responses are identical to those in the previous trial despite new information having been received; and anchoring, so that responses are biased by external, irrelevant cues. These biases are more prominent in children and older adults, whose cognitive resources are more limited on average and so are more likely to perseverate (Cepeda & Munakata, 2007; Ridderinkhof, Span, & Van Der Molen, 2002) and to anchor their responses (Munakata, Snyder, & Chatham, 2012).

Bruckner et al. (2025) developed a model incorporating MCMC algorithms to explain how these biases arise and why their magnitude changes across the lifespan. In their task, participants needed to place a bucket along a horizontal strip with 300 possible locations, so that it would catch reward bags dropped by a helicopter whose position was occluded by clouds. The drop point was determined by a draw from a Gaussian distribution with mean the helicopter position and fixed non-zero variance. In some trials, however, the helicopter changed positions to any point in the strip. Therefore, a bag drop in a given location could be due to typical fluctuations or to a change in the latent helicopter position.

According to their computational model, when people estimate the true location of the helicopter in a trial, they do so by producing samples from the posterior using the Metropolis–Hastings algorithm (implemented as sampler_mh() in the samplr package). They do so in chunks so that resource demands of the task are manageable. The posterior distribution they sample from is a mixture distribution, composed of a Uniform component (the belief over the new location if a change point has occurred) and a Gaussian component (the belief if a change point has not occurred); with a free parameter determining the sum weights (i.e., the participant’s a priori belief about the probability of a change point). Participants sample a chunk of *n* samples (a free parameter), then compare how different the mean of the samples is at predicting the data compared to the belief before sampling. If not very different (the tolerance for this is determined by the stopping criterion *c*, a free parameter), sampling stops, and the initial belief is used as a response (samples are discarded). Otherwise, the belief is updated to the mean of the samples and a new chunk of samples is drawn. Once a response is given, the prediction error is used to recompute the width of the prior. Changes in perseveration and anchoring across the lifespan can be explained by larger *n* values when cognitive resources are high.

If they had used the samplr package, their model could have been implemented using the sampler_mh() function to produce the samples (on our machine, this runs 31 times faster than their code). Their code for a trial update might have looked as follows (the functions calculate_posterior(), check_criterion(), and update_posterior_width() carry out processes we verbally describe above, but we do not define them for brevity):

**Figure:**
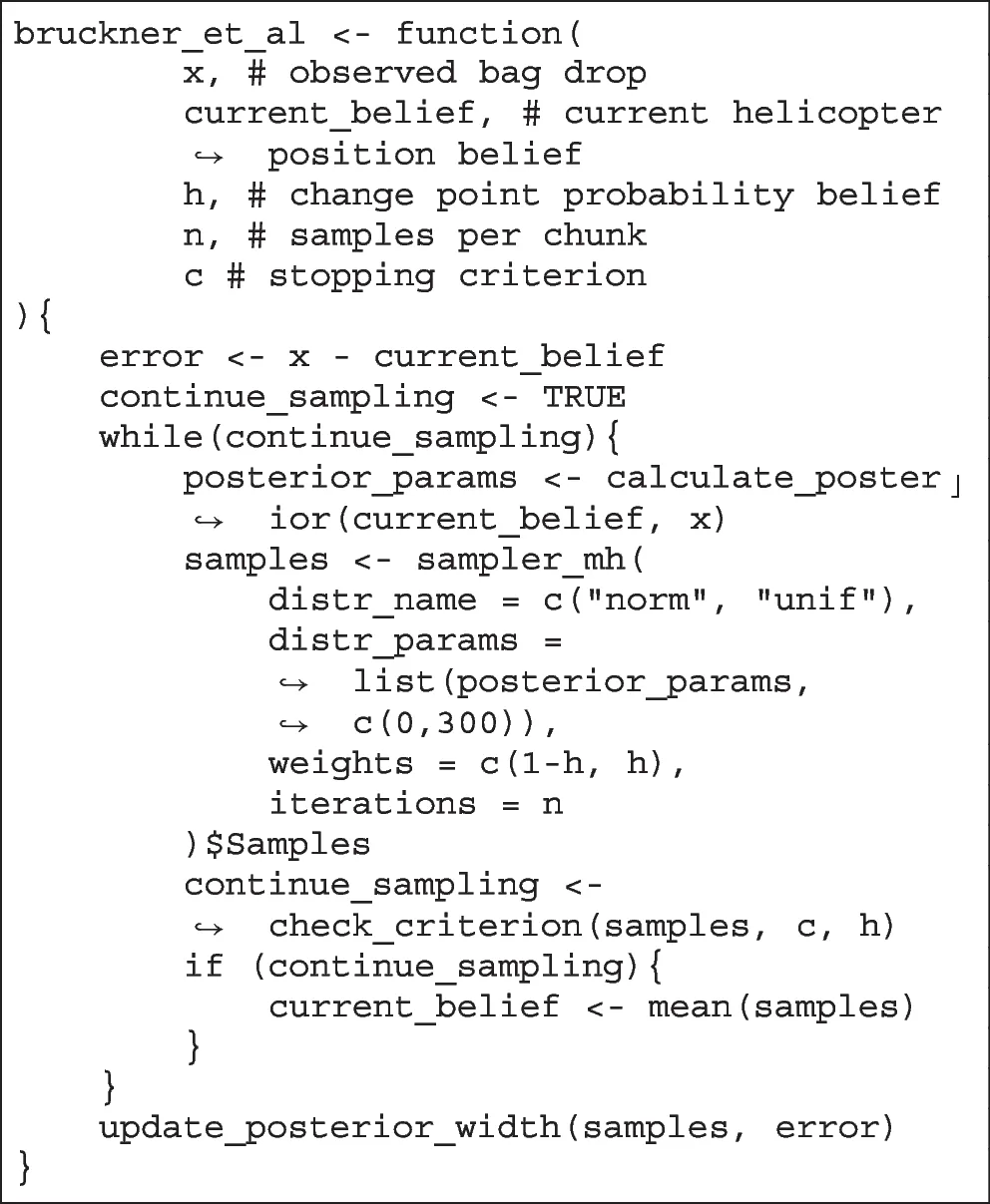

**Fig. 5:**
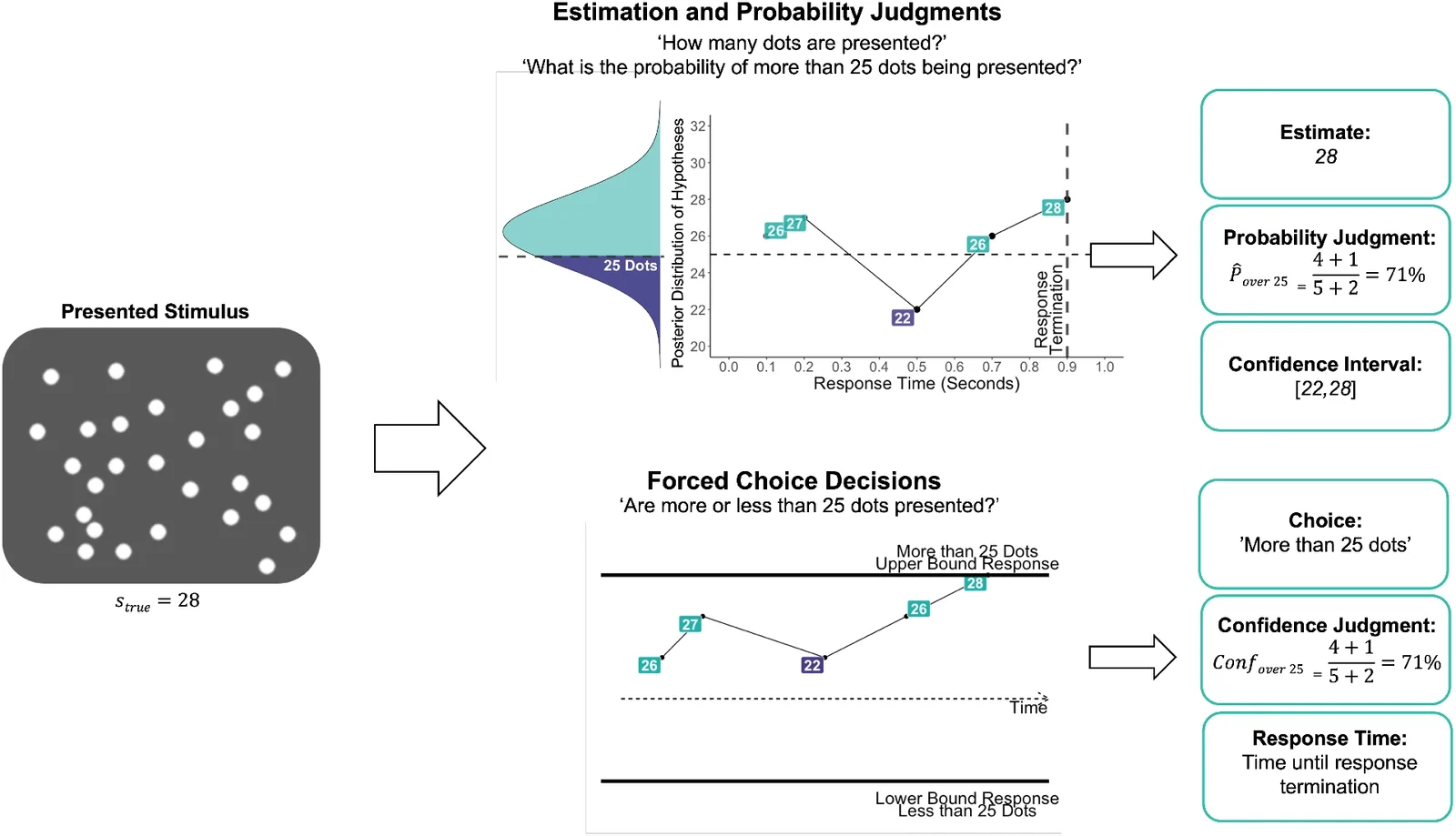
Schematic illustration of the autocorrelated Bayesian sampler (ABS, Zhu et al., 2024) applied to a numerosity task. The stimulus (*left*) is mentally represented as a Gaussian distribution, from which samples are drawn to support various task responses. These include: (1) estimates, (2) probability judgments, (3) confidence intervals, (4) choices, (5) confidence judgments, and (6) response times. *Note*. This figure is reprinted from “Noise in Cognition”, by Sanborn et al., 2025, Perspectives on Psychological Science, p.12. CC-BY

Of course, we have omitted some details here, as our objective was to show how samplr package functions can be used in a novel modeling task. Among details that we do not report here, we highlight that the model developed by Bruckner et al. (2025) can account for different learning rates depending on trial accuracy being more or less rewarded, for uncertainty being underestimated, for learning rates being affected by change-point probabilities, and for larger (or total) belief updating on catch trials where the helicopter is actually visible. The sampling component of the model, the Metropolis–Hastings algorithm, is implemented identically in both the samplr package and the samplr package.

### Choice accuracy and response times

The speed–accuracy trade-off is one of the key relationships between accuracy and response time in psychological tasks. Extensive research has demonstrated that individuals can prioritize accuracy by taking more time, or instead favor speed at the expense of accuracy (for a review, see Wickelgren, 1977). For instance, when accuracy is emphasized through instructions or incentives, individuals typically take longer to make judgments. Conversely, when speed is prioritized or decision time is constrained, judgments are often less accurate.

Within the framework of sequential sampling models that were developed in psychology, Ratcliff (1978) attributed this effect to the manipulation of the amount of information required for a judgment. More recent analysis using the drift diffusion model showed that this trade-off arises primarily from variations in the boundary separation parameter, followed by changes in the non-decision time and drift rates (Ratcliff & McKoon, 2023).

The autocorrelated Bayesian sampler (ABS; Zhu et al., 2024), a cognitive model in the samplr package, imports this successful explanation into a statistical sampling framework. As illustrated in Fig. 5, ABS is a sequential sampling model assuming people draw discrete and autocorrelated samples from a distribution, which represents signal strength, to make judgments and decisions. For two-alternative forced-choice tasks, ABS employs a relative stopping rule: sampling continues until the accumulated difference in evidence between the two alternatives exceeds a decision threshold.

Within this framework, the speed–accuracy trade-off arises from variations in the threshold level required by the task. Higher thresholds demand more evidence, resulting in longer response times but greater accuracy; lower thresholds require less evidence, producing faster but less accurate responses.

The following code demonstrates how to run ABS simulations to reveal this effect. ABS models were programmed using the R6 object-oriented programming system (Chang, 2025). Therefore, the first step in the simulation is to initialize the model using $new(), where users specify how the internal sampler operates, how samples are mapped to response time, and what the distribution to sample from is (distr_name specifies the name of the distribution and distr_params provides its parameter values). Currently, ABS uses MC^3^ as its sampler, a Markov chain Monte Carlo algorithm that generates new samples by adding normally distributed, unbiased noise to the previous item (width defines the width of the noise distribution). It runs multiple replicas, which swap positions stochastically (n_chains sets the number of chains; see more details in the Estimates section below, and in the Appendix). Response time is calculated as the sum of non-decision time (drawn from a uniform distribution U[nd_time, nd_time+s_nd_time]) and decision time (a random draw from an Erlang distribution Erlang(k,λ), where *k* is the number of samples drawn by the sampler and λ is provided by the user [lambda]).

After the initialization, users can use the $simulate() function to run the simulation. For this example, where a relative stopping rule is used, users need to provide information about how different the stimuli are (discrim), and where the boundary dividing support for one or the other option lies (dec_bdry). Finally, the key parameter here is the evidence threshold (delta): if large (e.g., delta = 10), sampling will be longer and more accurate. Conversely, when speed is prioritized, a smaller threshold is sufficient (e.g., delta = 3), resulting in shorter sampling durations. The results of the simulation can be accessed via $sim_results.

**Figure:**
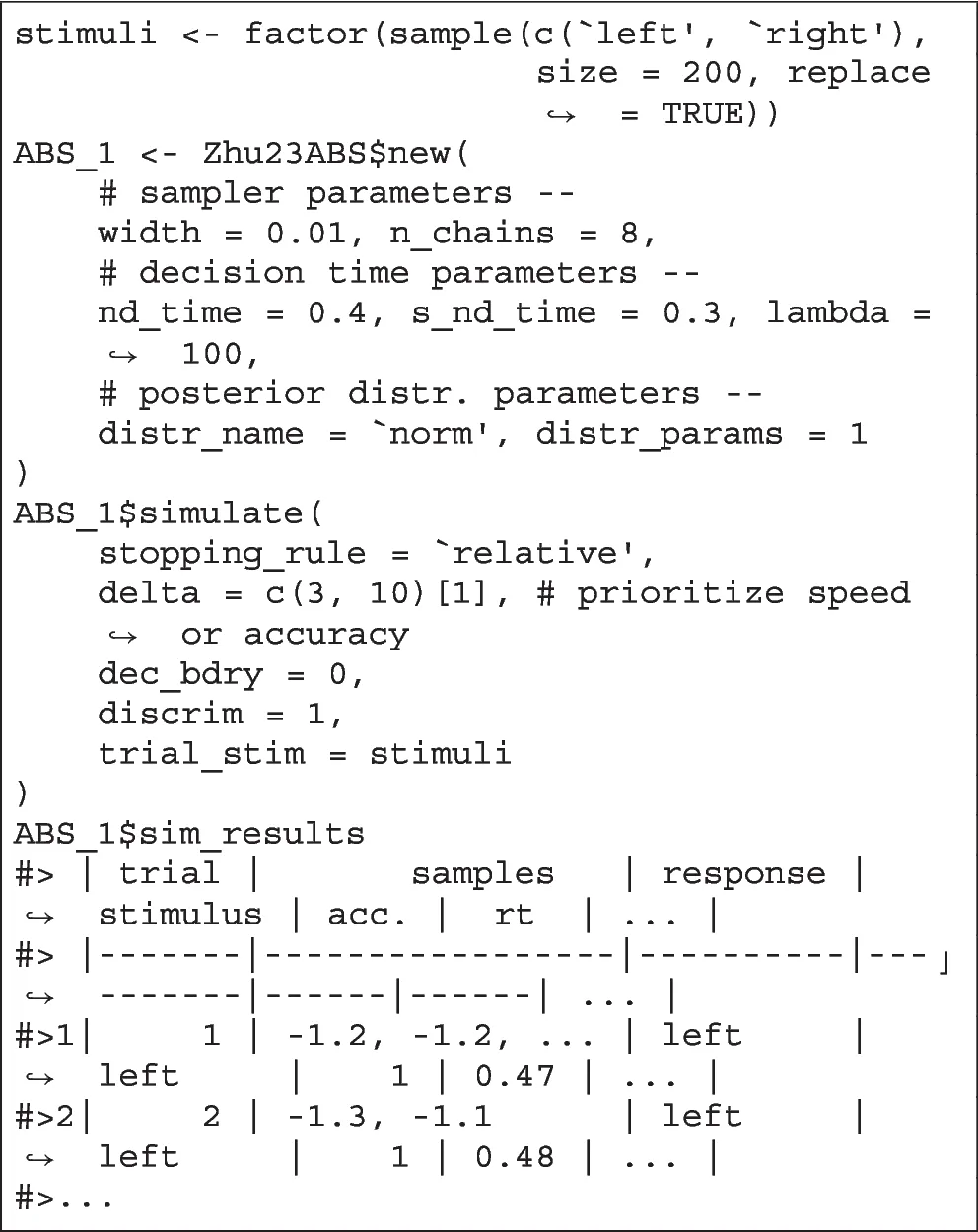

**Fig. 6:**
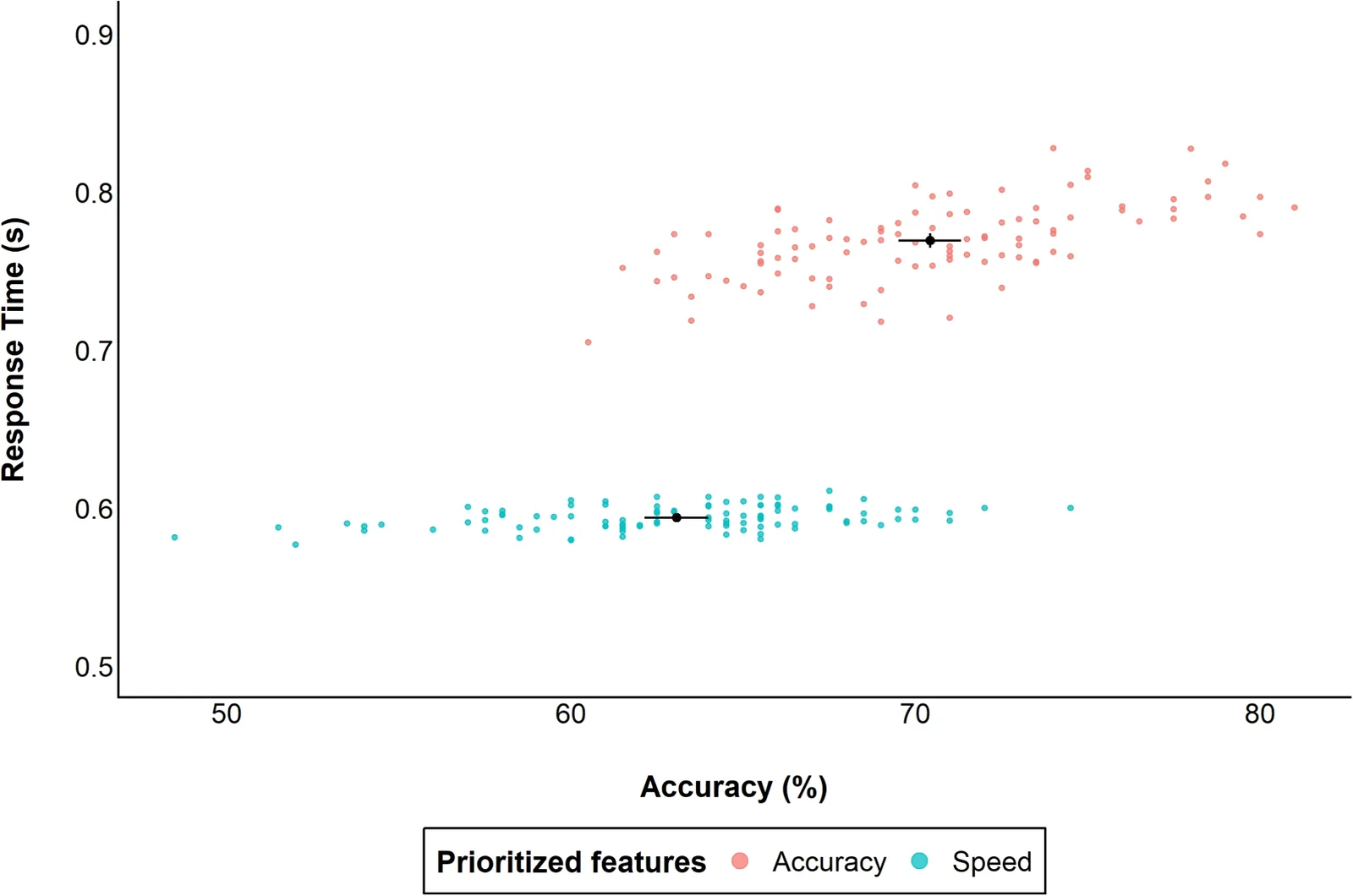
ABS prediction of the speed–accuracy trade-off. When the evidence threshold is small (*in blue*), responses are fast but exhibit reduced accuracy. Conversely, an increased evidence threshold is associated with higher accuracy and response times. The *black dots* mark the mean values of accuracy and response time. The *black bars* denote the 95% confidence intervals on both axes. ABS = autocorrelated Bayesian sampler

Figure 6 illustrates ABS simulations of accuracy and mean response time using the samplr package, where only the evidence threshold varies while all other parameters remain fixed. These simulations show that higher accuracy is consistently associated with longer response times.

### Metacognition: Confidence intervals

Subjective belief can be elicited through single-point estimates, but also by asking participants to provide a confidence interval with a pre-stated probability. For instance, participants might be asked to provide a 90% confidence interval for Finland’s population. Research indicates that people often exhibit overconfidence, producing over-precise estimates in both general knowledge and sensory discrimination tasks (Juslin, Winman, & Olsson, 2003; Juslin et al., 2007; Moore & Healy, 2008). In other words, their reported confidence intervals are narrower than they should be.

ABS follows the approach of Juslin, Winman, and Hansson (2007) and assumes that, when providing estimates, people draw samples from a distribution and terminate the process once a fixed number of samples has been collected. To produce these within an ABS framework (as opposed to via sampling algorithms directly, as explained above), the procedure is very similar to producing choices, with the exception that results are simulated using a “fixed” stopping rule, where they need to provide the number of samples wanted (n_sample):

**Figure:**
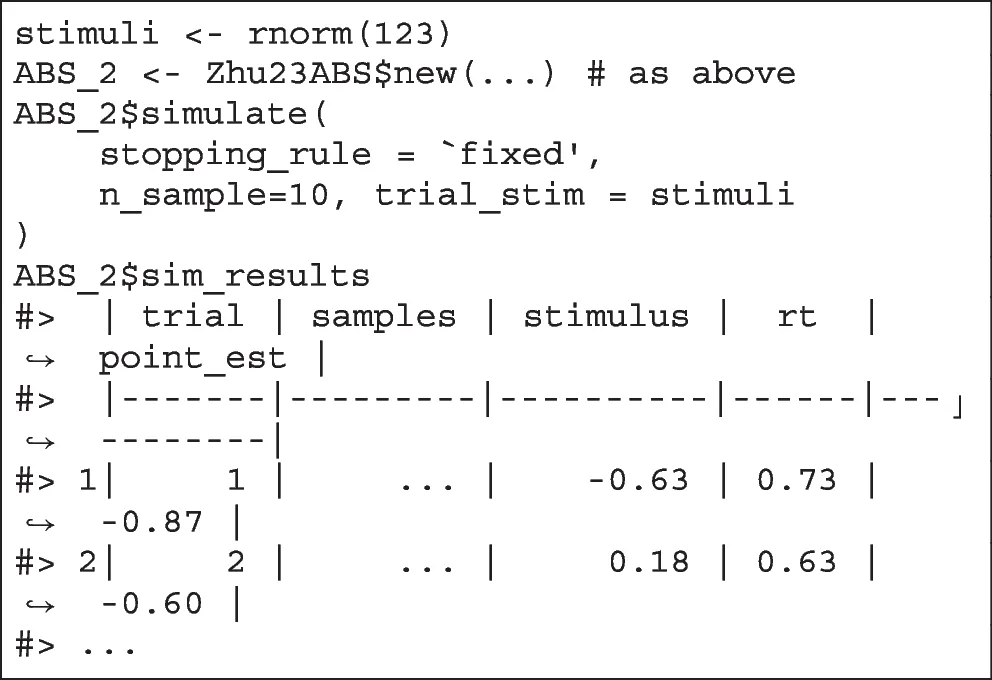

**Fig. 7:**
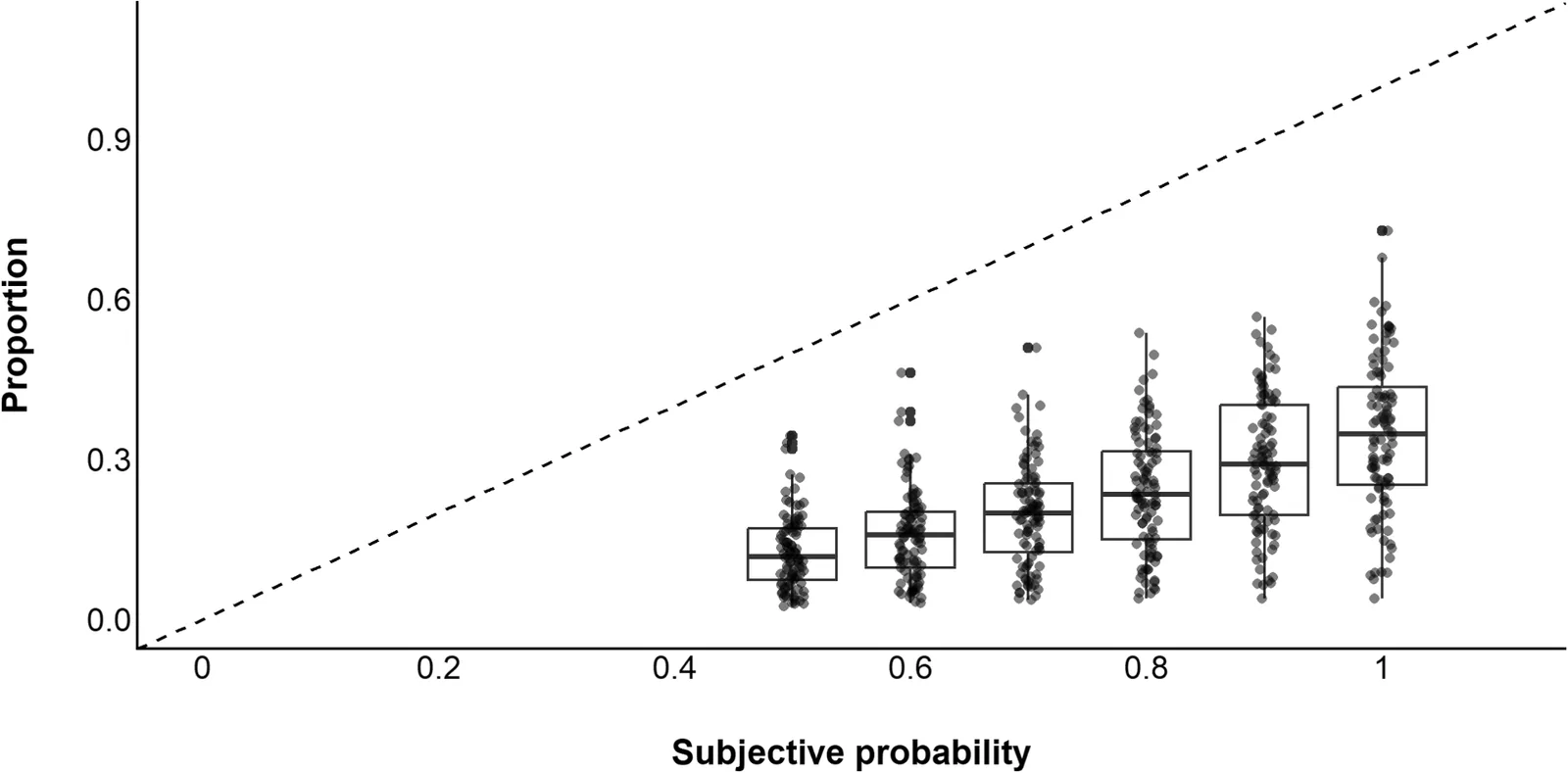
ABS prediction of the over-precision effect. The subjective probability represents the confidence level requested from ABS, while the proportion reflects the probability of the true distribution falling within the interval provided by ABS. The *dashed line* illustrates perfect calibration. ABS consistently predicts over-precise confidence intervals (dots) across all levels of subjective probability. ABS = autocorrelated Bayesian sampler

**Fig. 8:**
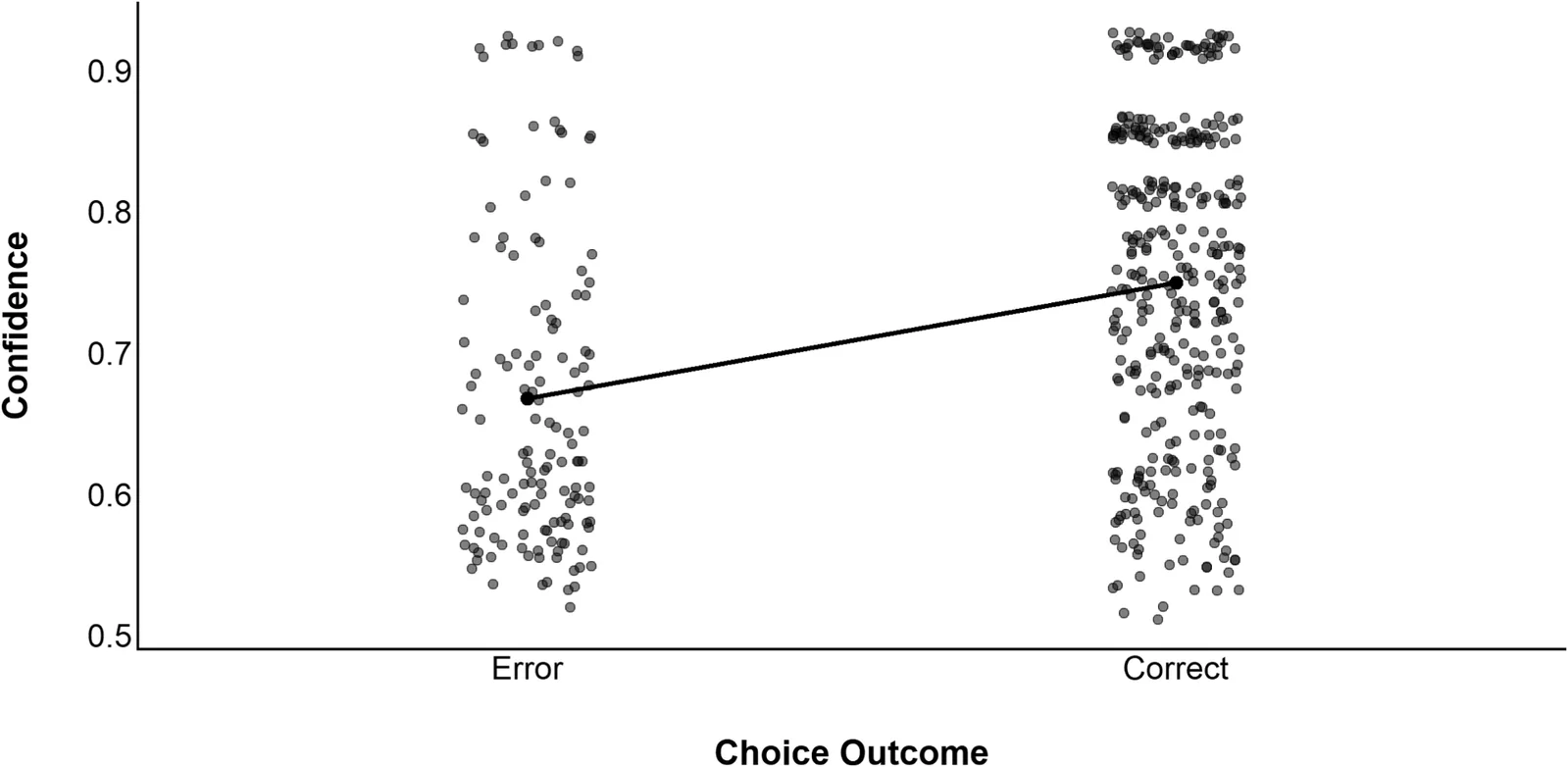
ABS prediction of the resolution of the confidence effect: Confidence levels are higher for correct responses than for errors. Each *dot* represents the confidence rating for a single trial. ABS = autocorrelated Bayesian sampler

**Fig. 9:**
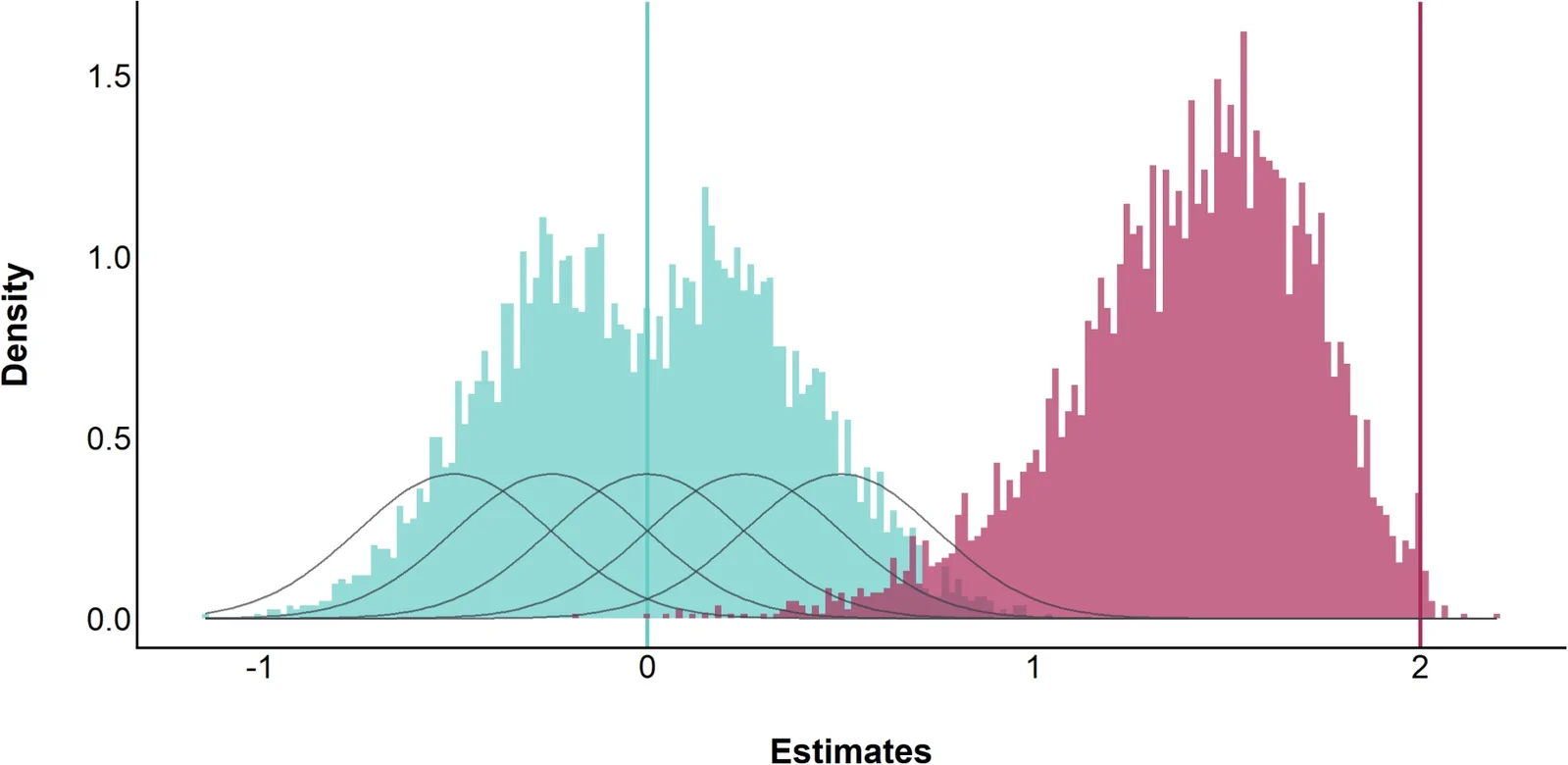
ABS prediction of the anchoring (*in green*) and repulsion effects (*in red*). The *black bell curves* represent the distribution of stimuli. Estimates are pushed away from a nearby comparison value (indicated by the *green line*, with the *green bars* showing the distribution of estimates) but pulled toward a distant comparison value (indicated by the *red line*, with the *red bars* showing the distribution of estimates). ABS = autocorrelated Bayesian sampler

Confidence intervals are then constructed based on the quantiles of the samples drawn. When the number of samples is limited, the distributional tails are typically underrepresented, leading to a true proportion that is lower than the subjective probability (Fig. 7). Confidence intervals can be added to ABS outputs by calling the confidence_interval() function, with the pre-stated probability as the only parameter:

**Figure:**
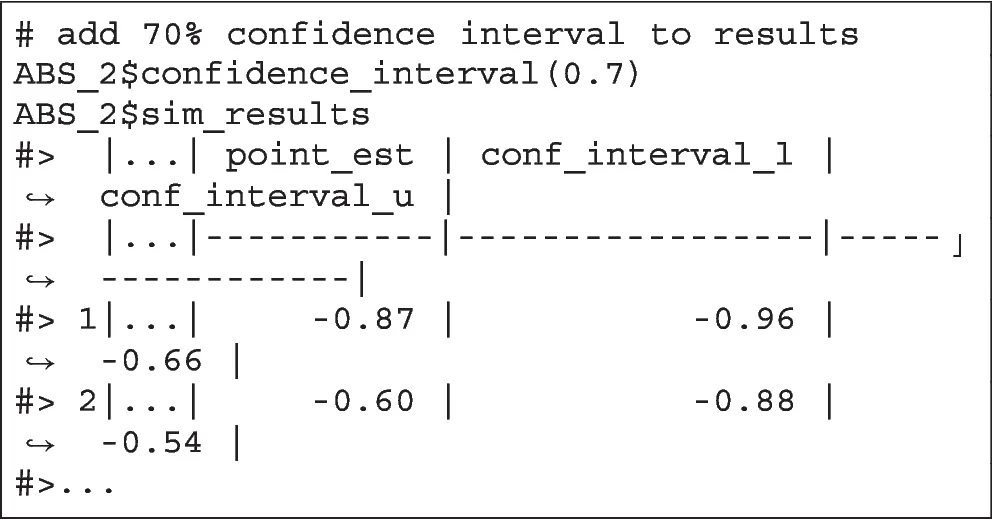

### Metacognition: confidence in decisions

Confidence can also be construed not as a range where an estimate may lie, but as the degree to which participants believe they made the correct choice. Research has consistently shown that average confidence ratings tend to be higher for correct responses than for incorrect ones, an effect often called the *resolution of confidence* (e.g., Ariely et al., 2000; Baranski & Petrusic, 1998; Vickers, 1979; Vickers & Packer, 1982). This confidence effect persists even when stimulus difficulty is held constant (Baranski & Petrusic, 1998) and when choice and confidence are elicited simultaneously from participants (Kiani, Corthell, & Shadlen, 2014; Ratcliff & Starns, 2009).

ABS assumes that individuals generate confidence estimates by calculating the proportion of samples drawn during choice that support each alternative. Because choices are made using a relative stopping rule where the support of samples for or against an option is autocorrelated, incorrect choices often occur when the evidence remains relatively balanced for an extended period, resulting in a slow error. In such cases, the total number of samples (i.e., the denominator in the confidence calculation) tends to be larger for errors than for correct responses (see Equation 9 in Zhu et al., 2024; Figure 8). For example, imagine a decision-maker who is asked whether a presented cloud of dots has more than 25 (as in Fig. 5). If their MCMC sampler samples around the 25-dot boundary, with several samples above and below 25 representing support and opposition for the ‘yes’ response, respectively, then their forced choice accumulation process will zigzag, taking a long time to reach one of the boundaries, and the proportion of samples for or against their choice will be closer to 50%. If, on the other hand, their MCMC sampler samples around 30, far from the boundary, successive samples will all be above 25, all supporting the ‘yes’ response, which will lead to a fast choice and high confidence.

In the samplr package, the confidence value for each choice is automatically stored in the confidence column of the $sim_results field. For the choices made above:

**Figure:**
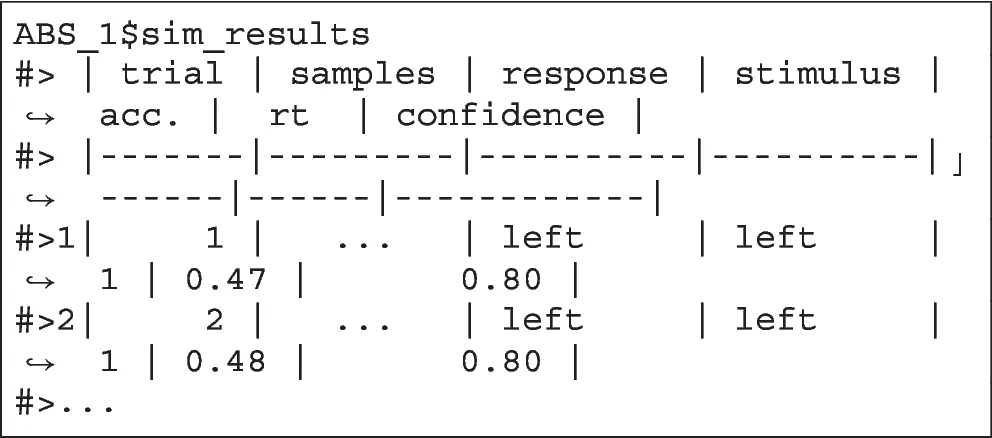

### Choices influence subsequent estimates: anchoring and repulsion effects

Human response modes are not independent of one another – instead they are known to interact. In cognitive judgments, estimates are often biased towards an initial value that is part of a choice, even when that value is irrelevant to the task (Epley & Gilovich, 2006; Tversky & Kahneman, 1974). This well-documented phenomenon, known as the *anchoring bias*, reflects the tendency for initial values to influence subsequent judgments (this is also seen in the Bruckner, Nassar, Li, & Eppinger, 2025 example shown above). However, more recent research in perceptual judgments, such as dot orientation or motion direction, has revealed a contrasting phenomenon: the *repulsion effect*, where estimates deviate away from the initial value (Jazayeri & Movshon, 2007; Luu & Stocker, 2018; Zamboni, Ledgeway, McGraw, & Schluppeck, 2016).

Spicer, Zhu, Chater, and Sanborn (2022) demonstrated that these seemingly opposed effects actually depend on the position of the initial value relative to the distribution of stimuli, and were similar across perceptual and cognitive stimuli. Specifically, estimates of the stimulus value are drawn toward the initial value if it is distant from the true value of the stimulus, replicating the anchoring effect, but are pushed away from the initial value if it is closer to the true value, replicating the repulsion effect.

These effects can be explained by the combination of an autocorrelated MCMC sampling algorithm that starts at the initial value and a relative stopping rule, two core components of ABS. In our simulation, we began with a set of distributions to represent the underlying stimulus distribution, illustrated by the black curves in Fig. 9. To run the ABS model on custom distributions, users should specify the custom_distr and custom_start arguments when initializing Zhu23ABS$new(). These allow the user to define both the custom stimulus distributions and the corresponding starting values for the sampling process.

**Figure:**
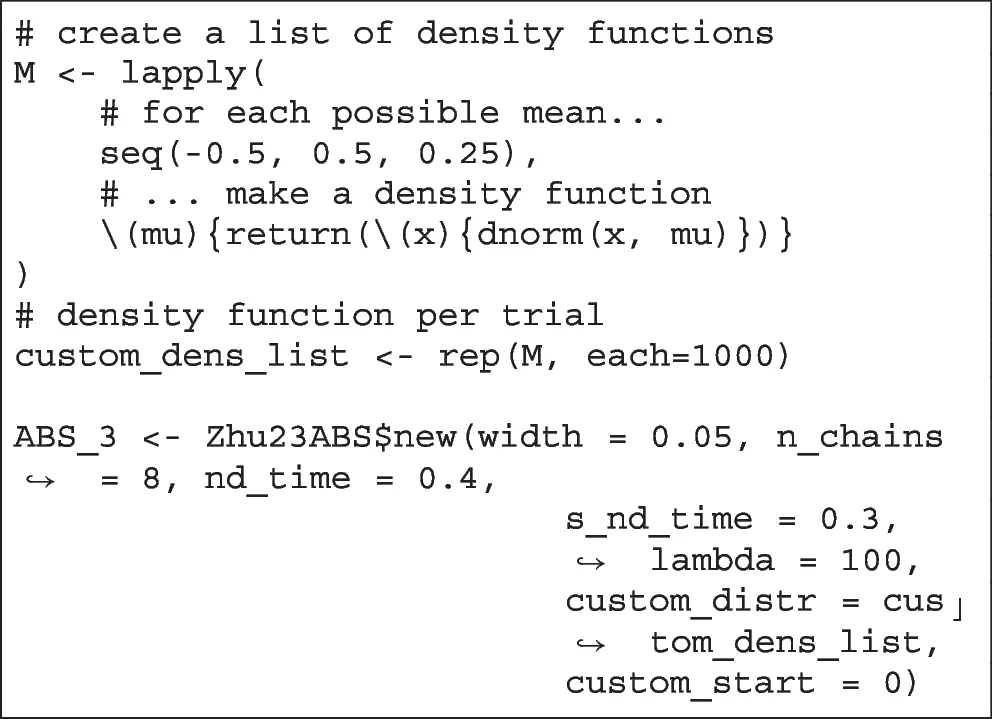

When the starting point and the decision boundary (indicated by the green line) are close to the stimulus distribution and effectively partition the distribution into two binary response regions, the relative stopping rule requires that the sampling process continue until a sufficient number of samples fall on one side of the boundary over the other. As a result, the final sample in the sequence, used as the estimate, tends to be away from the decision boundary, producing the repulsion effect. It is important to note that both fixed and relative stopping rules can yield point estimates, stored in the point_est column of the $sim_results field (when we showed outputs of the ABS_1 model above, we omitted this column for clarity).

On the other hand, if the decision boundary and the starting point (indicated by the red line) are distant from the stimulus distributions, the sampling process is driven by two opposing forces. While the process is drawn toward the stimulus distribution, the autocorrelation inherent in the sampling process acts as a counterbalancing force. Because nearly all samples fall on the same side of the decision boundary, the process tends to terminate before fully exploring the stimulus distribution, resulting in the anchoring effect.

Finally, one might also notice that the green histogram in Fig. 9 covers a narrower range than the full stimulus distribution (depicted by the black curves). This is because the sampling chains are autocorrelated and all begin at the green line, causing the final samples to cluster near the starting point, thus limiting their spread (i.e., anchoring is also occurring).

## Quantitative comparisons

So far, we have shown that the models here presented can qualitatively match patterns in human behavior. Researchers, however, need quantitative ways to compare models, and unfortunately, the models here presented are not tractable: no analytical solution exists for their likelihood function, and the computational cost of calculating it is too high. For this reason, we rely on likelihood-free approximations (for a recent review, see Cranmer, Brehmer, & Louppe, 2020, and see Palestro, Sederberg, Osth, Van Zandt, & Turner, 2018 and Sisson, Fan, & Beaumont, 2019 for more depth).

Likelihood-free inference is general in nature, and can in principle be used on any inference problem. All that is needed is that candidate models can simulate data, and all models in the samplr package can. For approximate Bayesian computation, which is the technique we will use below, researchers will also want a way to transform the data into a set of summaries. The samplr package offers some diagnostic functions (mostly starting with calc_*). For example, a user can run

**Figure:**
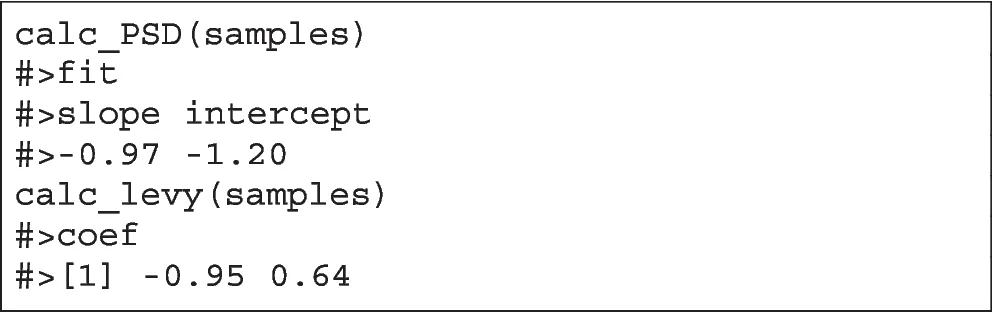

to obtain the power spectral density and Lévy flight coefficient of a sequence of samples. These measures have been used to characterize the long-range autocorrelations (Gilden, Thornton, & Mallon, 1995) of estimates and the heavy-tailed distributions of response times Rhodes and Turvey (2007) found in human estimates, respectively (see also Zhu et al., 2022). Crucially, however, the choice of summary statistics is domain-dependent, so depending on the specific application, the ones offered in the samplr package may need to be complemented by others.

Below, we give an example of how likelihood-free inference can be carried out, using approximate Bayesian computation (ABC). We perform quantitative analyses in two ways: first, via rejection ABC (Beaumont, Zhang, & Balding, 2002), which will serve to establish the reasoning behind how ABC works. Then, we will use a more recent version of approximate Bayesian computation, ABC with random forests (a machine learning algorithm; Van Zandt, 2012;  Pudlo et al., 2016; see Hastie, Tibshirani, & Friedman, 2009 for a general introduction to random forests).

### Example: quantitative comparisons in Castillo, León-Villagrá, Chater and Sanborn (2024a)

In Experiment 1 of Castillo et al. (2024a), participants generated a sequence of random heights (data from their experiments is available in the samplrData package; Castillo, Li, & Sanborn, 2024b, which is also available on CRAN). They were asked to imagine that every healthy adult in the UK had written their height on a piece of paper. In the *Gaussian* condition, all these pieces of paper had been put in a bag. In the *Uniform* condition, instead, the bag only contained unique values. Their task was to say one of the heights in the bag as if drawn, read, and restored each time. The authors compared participants’ data to six local sampling algorithms (those in Fig. 1) and another computational model of random generation (Cooper, 2016). For speed, we simplify their analysis here to evaluating the *Gaussian* condition against only the sampling algorithms in the samplr package.

The objective of the authors was to determine which model $m \in \mathcal {M}$ best described a given datum *y* (i.e., a sequence of heights); with the hurdle that the likelihood of the data given a particular model is intractable. The logic of ABC is to approximate the posterior $\pi (\mathcal {M}|y)$ by simulating data from the prior over the candidate models. One draws a random model from the prior over models, $m' \sim \pi (\mathcal {M})$, then simulates data from said model, $m' \rightarrow y'_{m'}$. If one discards all synthetic data that are not identical to the observed datum, then the distribution of surviving generating models will approximate the true posterior eventually, by the law of large numbers. For example, trying to infer how many six-sided dice were rolled to produce a sum of 11, one could repeatedly roll *n* dice (with *n* drawn from a prior n∼[2,10]), then only tally the generating *n* if the roll added exactly to 11. This would, given enough rolls, approximate the correct posterior eventually.

In practice, the requirement of models needing to produce synthetic data identical to the one observed is too strict, leading to prohibitively expensive inference as too many simulations are required. Instead, some of these requirements are relaxed, in an exercise balancing accuracy and computation (Sisson et al., 2019): First, summary statistics instead of full datasets are used, so that the posterior distribution $\pi (\mathcal {M}|y)$ is replaced by the partial posterior $\pi (\mathcal {M}|s_y)$ where $s_y = S(y)$ and S(·) is a function generating a vector of summary statistics from the data. If *S* is *sufficient* for $\mathcal {M}$, i.e., it captures all the information in the data that is relevant to the estimation of $\mathcal {M}$, then no loss of information occurs at this stage (Didelot, Everitt, Johansen, & Lawson, 2011). Second, a tolerance ϵ is defined, so that synthetic data are only rejected if the distance between $s_y'$ and $s_y$ is greater than ϵ.

The choice of summary statistics and tolerance needs to be integrated into a larger Bayesian workflow that ensures inferences are appropriate (as will all Bayesian methods; see Gelman et al., 2020; Schad, Betancourt, & Vasishth, 2021). For example, before fitting the data one must ensure that parameter priors for each model produce sensible data, or that the fitting procedure recovers the generating models and/or parameters in silico, tweaking the choice of summary statistics and tolerance in case it does not. Failing to perform these steps can lead to incorrect inferences: for example, as with all Bayesian selection methods, this procedure is sensitive to the choice of parameter priors for models. One model might be disfavored if its prior over parameters is not well specified (e.g., our dice model would perform poorly at matching a dice sum of 11 if the prior over *n* was from five to ten). If this is a potential worry (e.g., because experimenters are using models they are not very familiar with), a potential solution is to use part of the data to derive good parameter distributions for each model (see how to perform parameter estimation with this approach below), then use the rest of the data in the model selection exercise (O’Hagan, 1995).

To return to the example of estimating which model best reproduces the sequences of random heights people produced, the authors first generated synthetic data with their models (here we produce 10,000 simulations per model). The sampling algorithms sampled from a normal distribution with mean μ=176.4 and standard deviation σ=12 (derived from the data). The prior over models was uniform, and the prior over parameters is defined in Table 2, with simulation parameters being randomly drawn from this prior. The summary statistics used were those in Table 1. To reiterate, the choice of summary statistics when using this method is important as ideally they would be sufficient. However, this ideal is difficult to achieve in practice, partly because whether a set of summary statistics is sufficient is unknowable. Typically, the choice of summary statistics is based on experimenter knowledge only, although efforts to automate part of this process have been developed recently (Fearnhead & Prangle, 2012; Valentin, Castillo, Sanborn, & Lucas, 2024).

**Table 2:** Prior over parameters for models used in quantitative model comparison example, following Castillo et al. (2024a)

Parameter Name	Value	Used by
Proposal Width	U∼(0,50)	MH,MC^3^
Temperature Increase	U∼(1,4)	MC^3^,MCHMC, MCREC
Number of Chains	4, 5, 6	MC^3^,MCHMC, MCREC
Swap All	*yes*, *no*	MC^3^,MCHMC, MCREC
Trajectory Length	1,2,⋯,100	HMC,REC,MCHMC,MCREC
Recycling Rate	U∼(0,1)	REC, MCREC

Once the matrix of summary statistics and generating models is available, observed and synthetic summary statistics should be standardized (using the mean and SD from the synthetic data), so that all summaries are weighted equally. Following (Biau, Cérou, & Guyader, 2015), we use a convenience tolerance $\epsilon ^*$ so that only 10% of simulations are kept, and we find that MCREC is the model with the largest support, with a $BF_{10} > 400$ over the next best model.

Whilst powerful, rejection ABC is not without limitations: it requires a considerable volume of simulations to approximate the posterior accurately, and it is sensitive to the choice of summary statistics. For example, the set we use here (Table 1) is not a set of orthogonal statistics, and it could be argued that some aspects of the sequence, like the distance between items, are evaluated by more than one summary statistic (like *Adjacencies* and *Distances*) whereas other aspects, like the tendency to change from a descending to an ascending run or vice versa, are only measured by one (*Turning Points*). Therefore, if all summary statistics are weighted equally (as we do), then models that do not replicate the distances between items well will be penalized more strongly when computing the posterior.

Pudlo et al. (2016) developed an ABC technique that addresses these limitations. Instead of evaluating the matrix of summary statistics directly, they run it through a machine learning classifier, a random forest, that learns the non-linear relationships between summary statistics that best predict which model produced the synthetic data, therefore weighing each summary statistic as a function of its predictive value, and needing fewer simulations than rejection ABC. Once trained with the simulations, the observed data serve as the test data that random forests allocate to generating models.

**Fig. 10:**
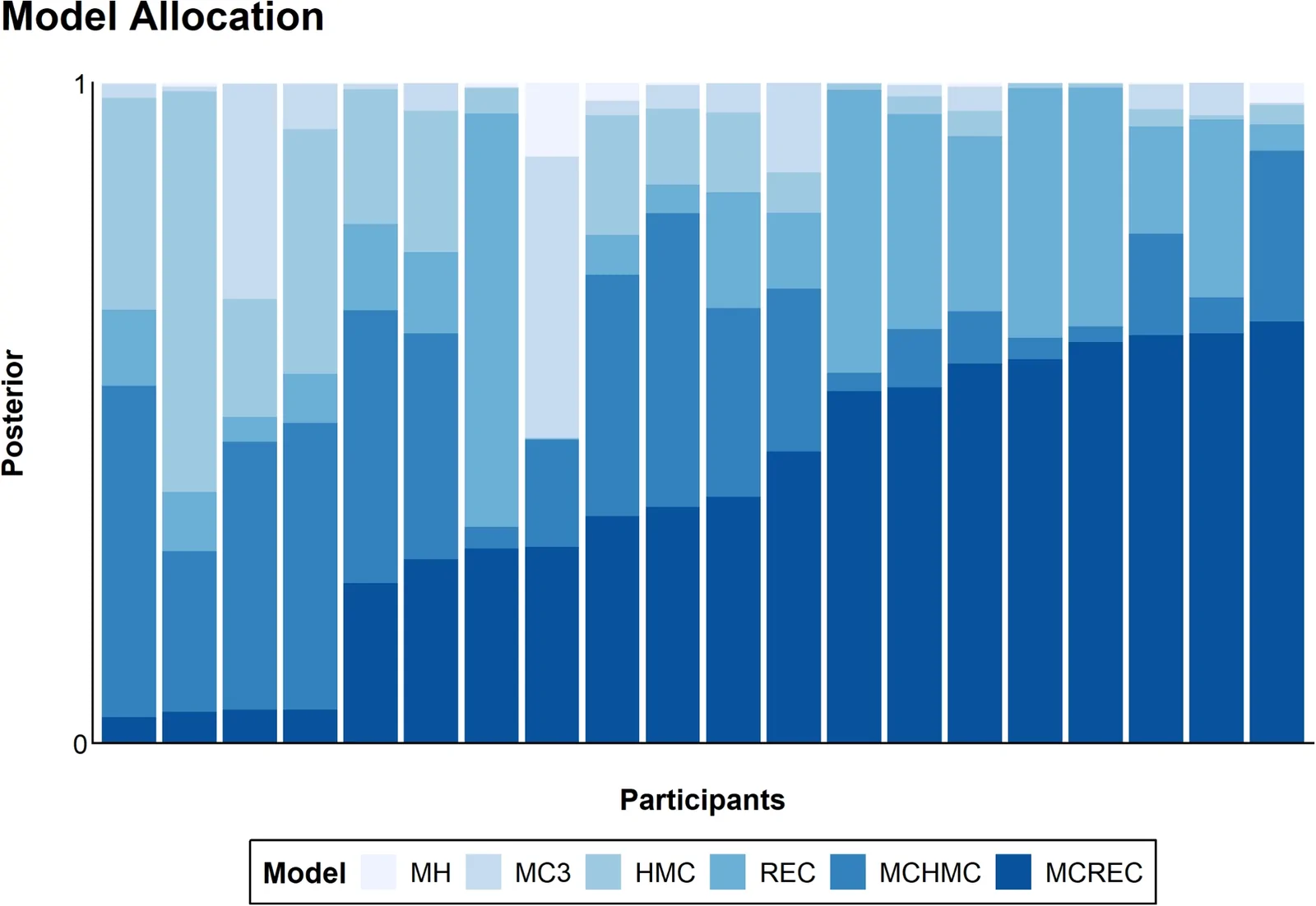
Model selection results for the Gaussian condition in Experiment 1 of Castillo et al. (2024a), with *bar lengths* showing the posterior probability of models. Each *column* represents a different participant

For convenience, we use the abcrf package here (Pudlo et al., 2016) to perform inference on the Castillo et al. (2024a) data. We reuse the simulations and summaries generated above, then train the random forest using the abcrf() function their package provides. Once the forest has been trained, a useful metric is the out-of-bag error: the percentage of trees that haven’t seen a given simulation during training and misclassify it during this checking step. In our case, with the abcrf default settings, this is 28.27%.

The final step is to ask the random forest classifier to allocate models to the observed data. For each tree, the posterior distribution over models is the relative frequency of model labels in the training data for which the same classification was made. In other words, the tree classifies the data and then remembers the proportion of true generating models when it reached the same leaf during training. The posterior distribution of the forest is the average of tree posteriors, which is accurate given sufficient trees. Using this approach, the support for MCREC was $BF_{10} > 10^3$ over the next best model. We show model classification results for each participant in Fig. 10.

Parameter estimation follows a very similar procedure for rejection ABC: first, simulations are generated by drawing parameter vectors from the prior over parameters, and after rejecting all simulations for which the vector of summary statistics is too far from the data, the distribution of surviving parameters approximates the posterior over parameters. For a treatment of parameter estimation with random forests, see Raynal et al., 2019 (also implemented in the abcrf package).

## Discussion

We presented the samplr package, an R package available in the CRAN repository that contains several statistical sampling algorithms, as well as cognitive models using sampling, to compare to human data – in addition to many diagnostic tools to aid this process. We have shown how these cognitive explanations can match a wide range of interesting qualitative observations of human behavior – be it errors in probability judgments; relationships between choice and speed or confidence; starting point influences; or the characteristic long-range structure of repeated estimates. Also, we have presented two of the many ways that these models can be evaluated quantitatively, giving a short example of how to use rejection-based approximate Bayesian computation as well as how to use random forests for ABC as proposed by Pudlo et al. (2016).

We invite researchers studying different psychological and behavioral phenomena to try these approaches, and we provide support with the commented examples that supplement this paper, showing how our figures were produced. As a potential starting point, datasets collected by our lab – which have already been evaluated using these approaches – are available in the samplrData package (Castillo et al., 2024b), also available on CRAN.

After all, an exciting feature of these statistical sampling algorithms is how general they are in purpose: they have been applied as explanations of phenomena from low-level perception (Gershman, Vul, & Tenenbaum, 2012) to causal reasoning (Bramley, Dayan, Griffiths, & Lagnado, 2017; Davis & Rehder, 2020). What is more, they are not limited to very simple representations as other cognitive models might be: for example, in an experiment involving descriptions of people, experimenters could transform the text participants read into a semantic vector space (e.g., as done by Bhatia, 2017), and then use statistical sampling algorithms to sample from that space with the probabilities given by a complex neural network (Sanborn, Yan, & Tsvetkov, 2024).

A particularly exciting possibility is that potentially there is a single sampling approach that can explain a wide range of judgments and behaviors. The ABS uses a single sampling process and simple mapping rules to explain probability judgments, choices, response times, confidence judgments, estimates, and confidence intervals, and even interactions between these response modes (Zhu et al., 2024). Being able to quantitatively fit the same model to such a wide range of judgments will provide much greater insight into human perception and cognition than examining separate tasks and response modes in isolation, and potentially make much more powerful predictions.

We hope that by making this set of functions available, we can facilitate using these approaches as well as make their implementations homogeneous; akin to how software packages implementing sequential sampling algorithms have done so (Singmann et al.,^2018^; Vandekerckhove & Tuerlinckx, 2008; Wiecki et al., 2013). Importantly, the samplr package is free and open software in active development, and we welcome requests for new features as well as invite other researchers to expand the software.

## Figures and Tables

**Algorithm 1:**
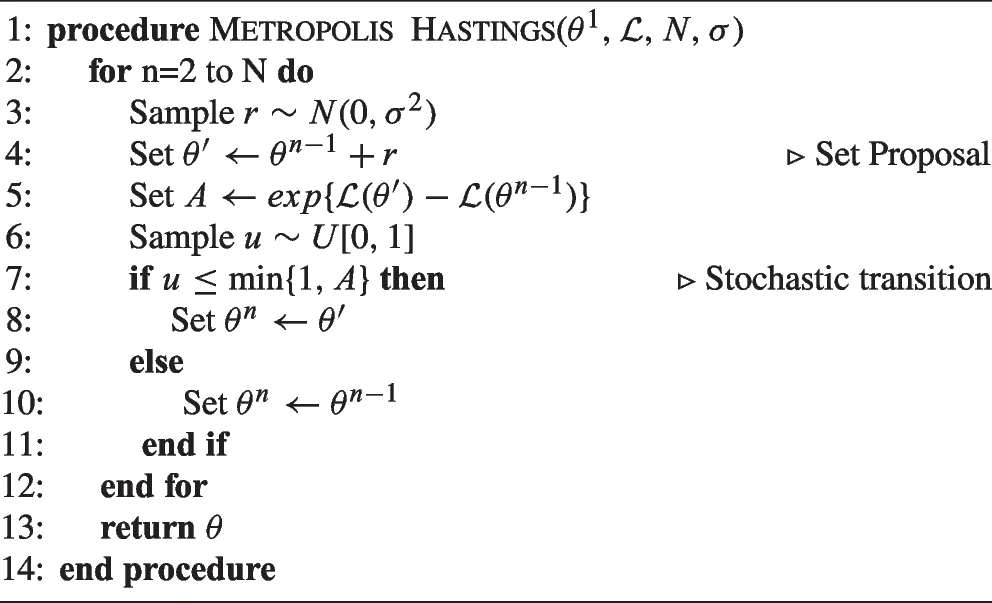
Metropolis–Hastings

**Algorithm 2:**
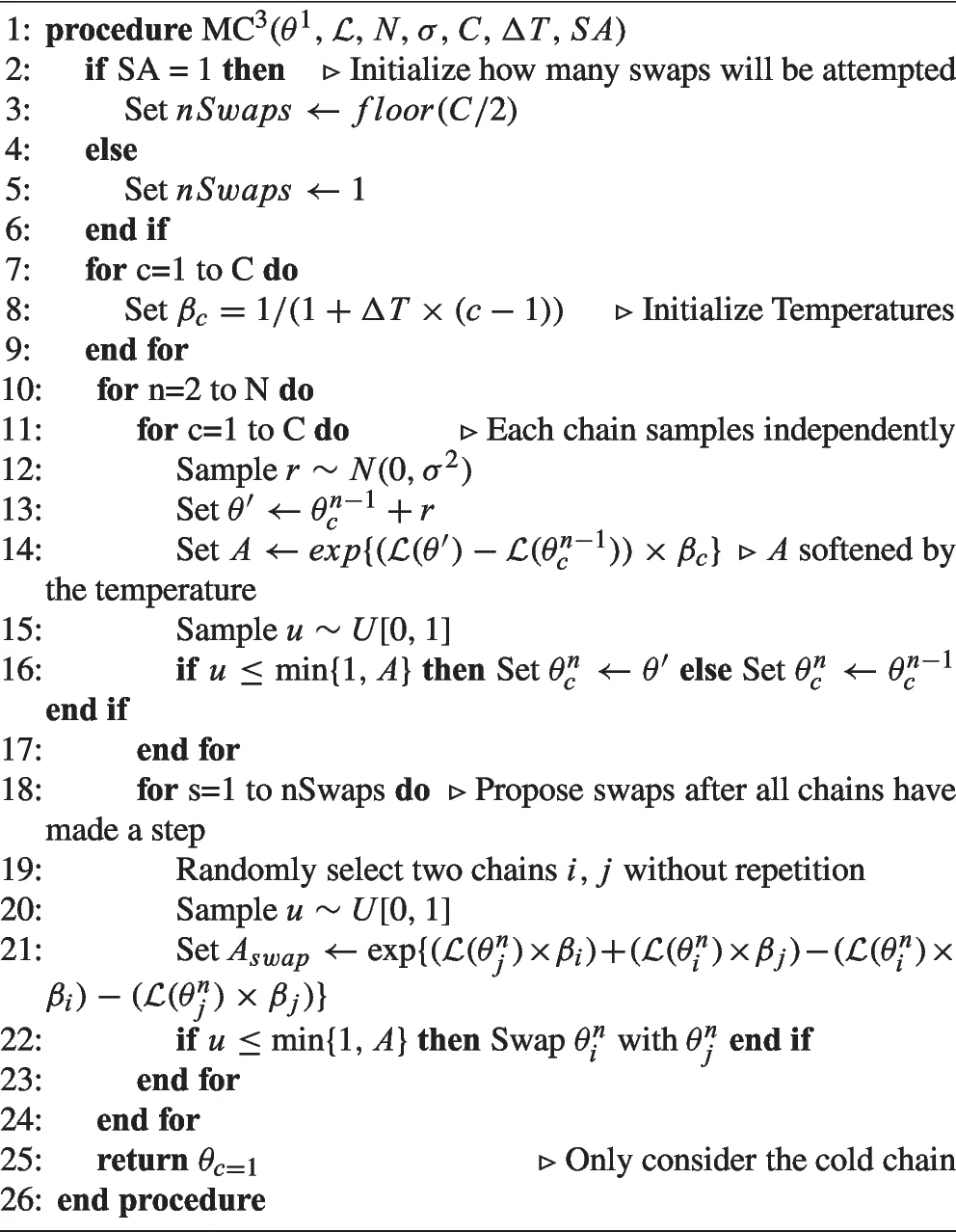
Metropolis-coupled MCMC (MC^3^)

**Algorithm 3:**
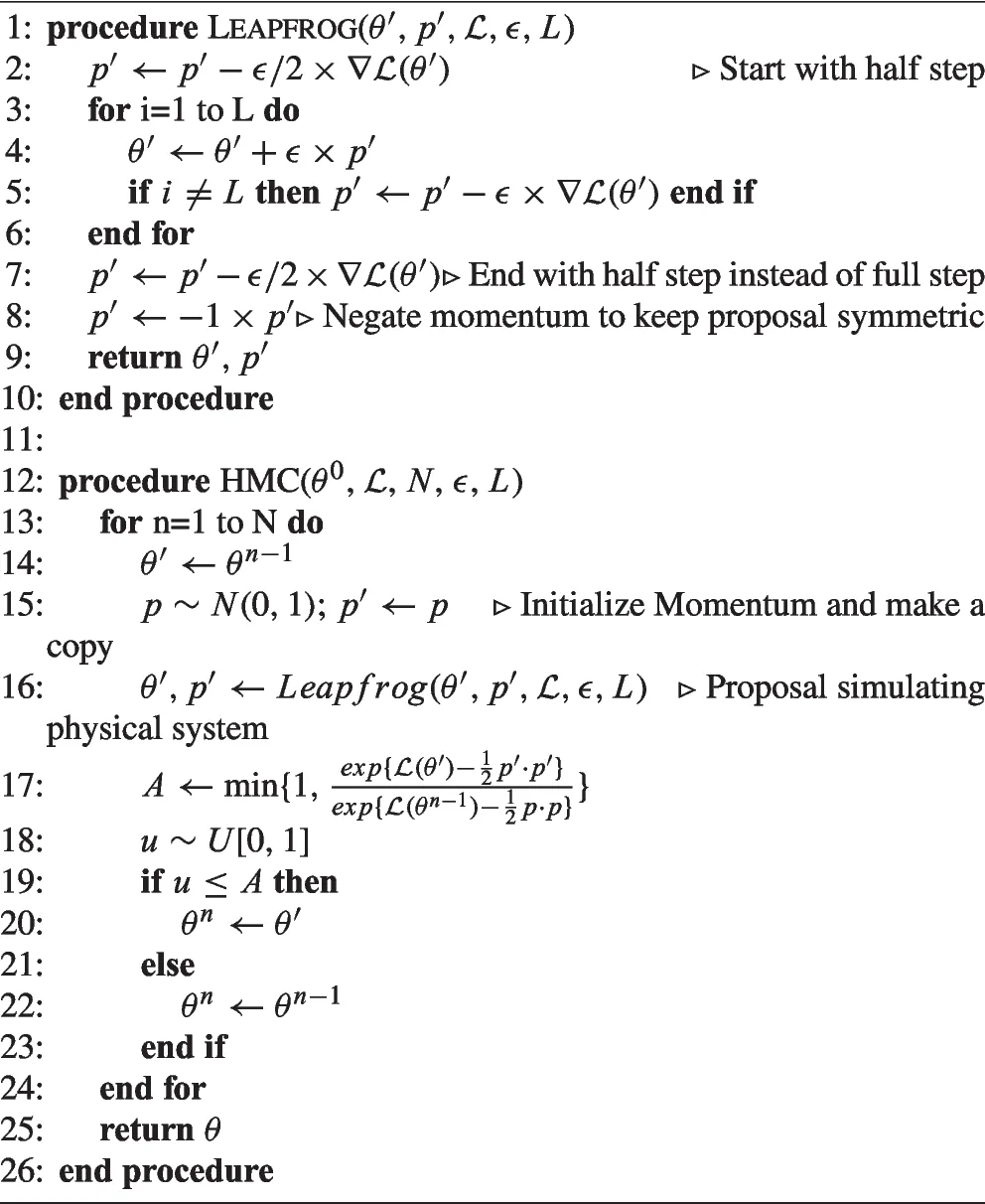
Hamiltonian Monte Carlo

**Algorithm 4:**
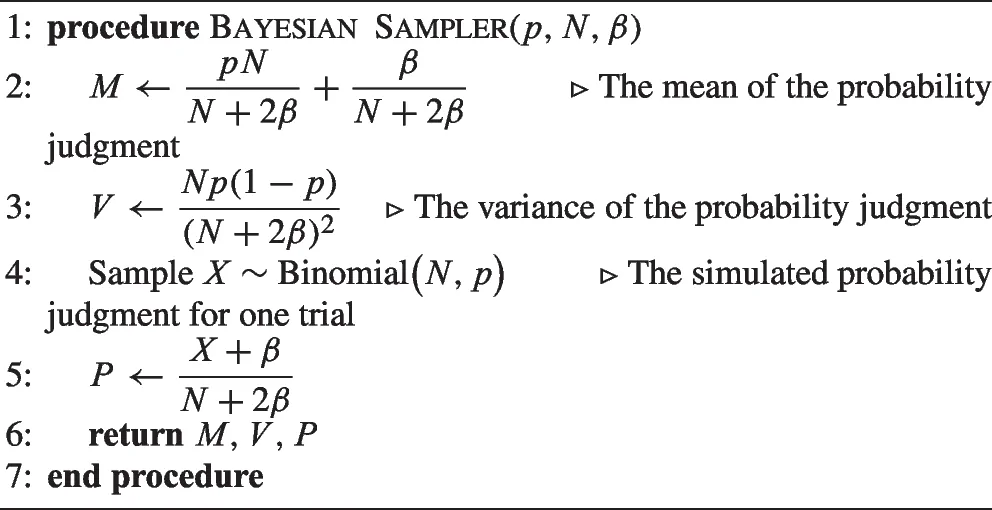
Bayesian sampler

**Algorithm 5:**
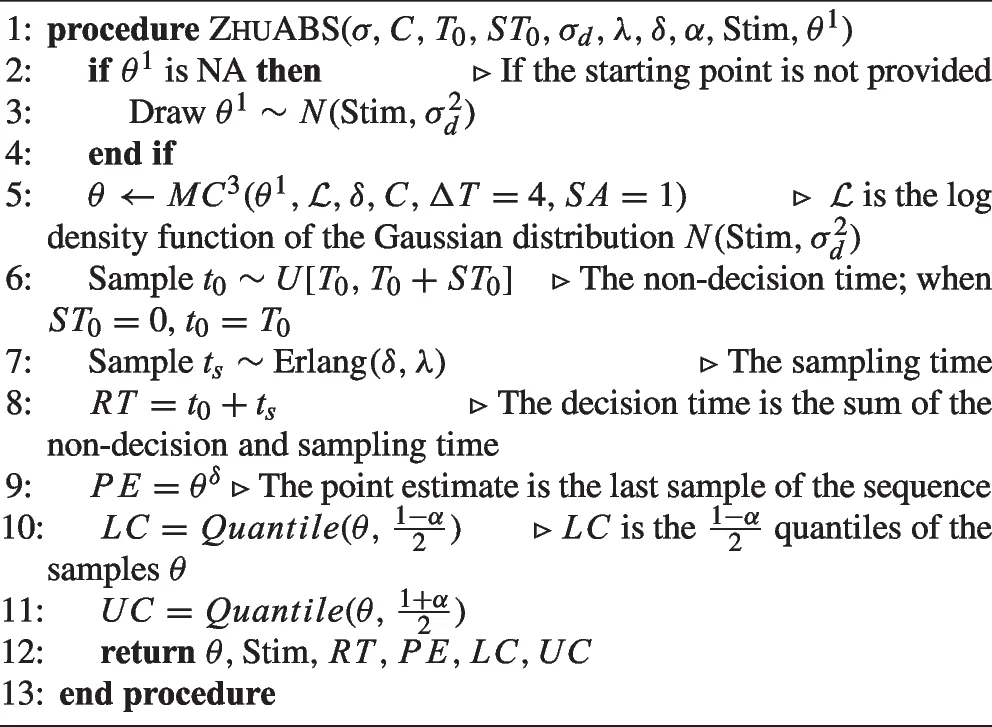
Autocorrelated Bayesian sampler with fixed stopping rule for one trial

**Algorithm 6:**
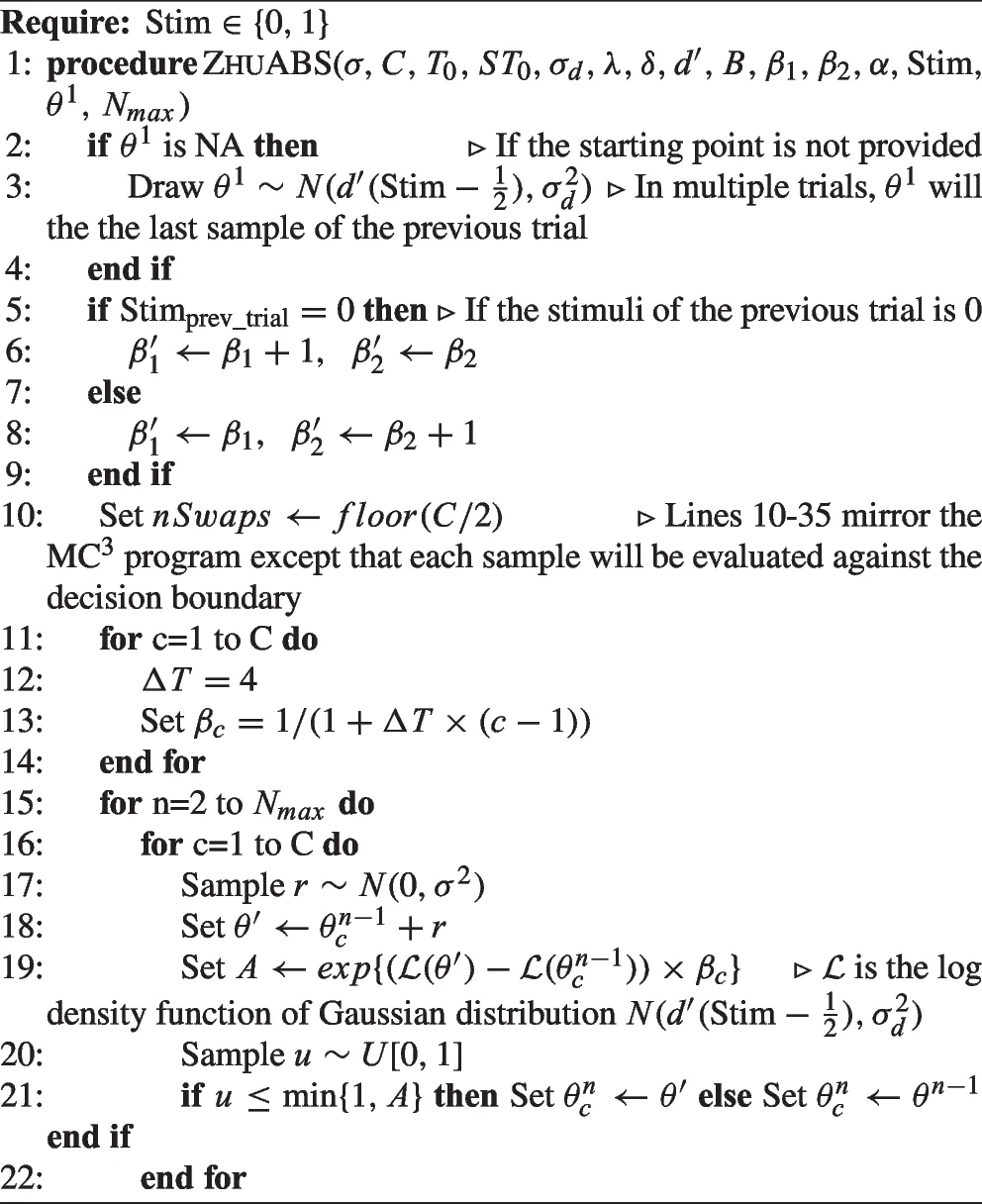
Autocorrelated Bayesian sampler with relative stopping rule for one trial

**Algorithm 7:**
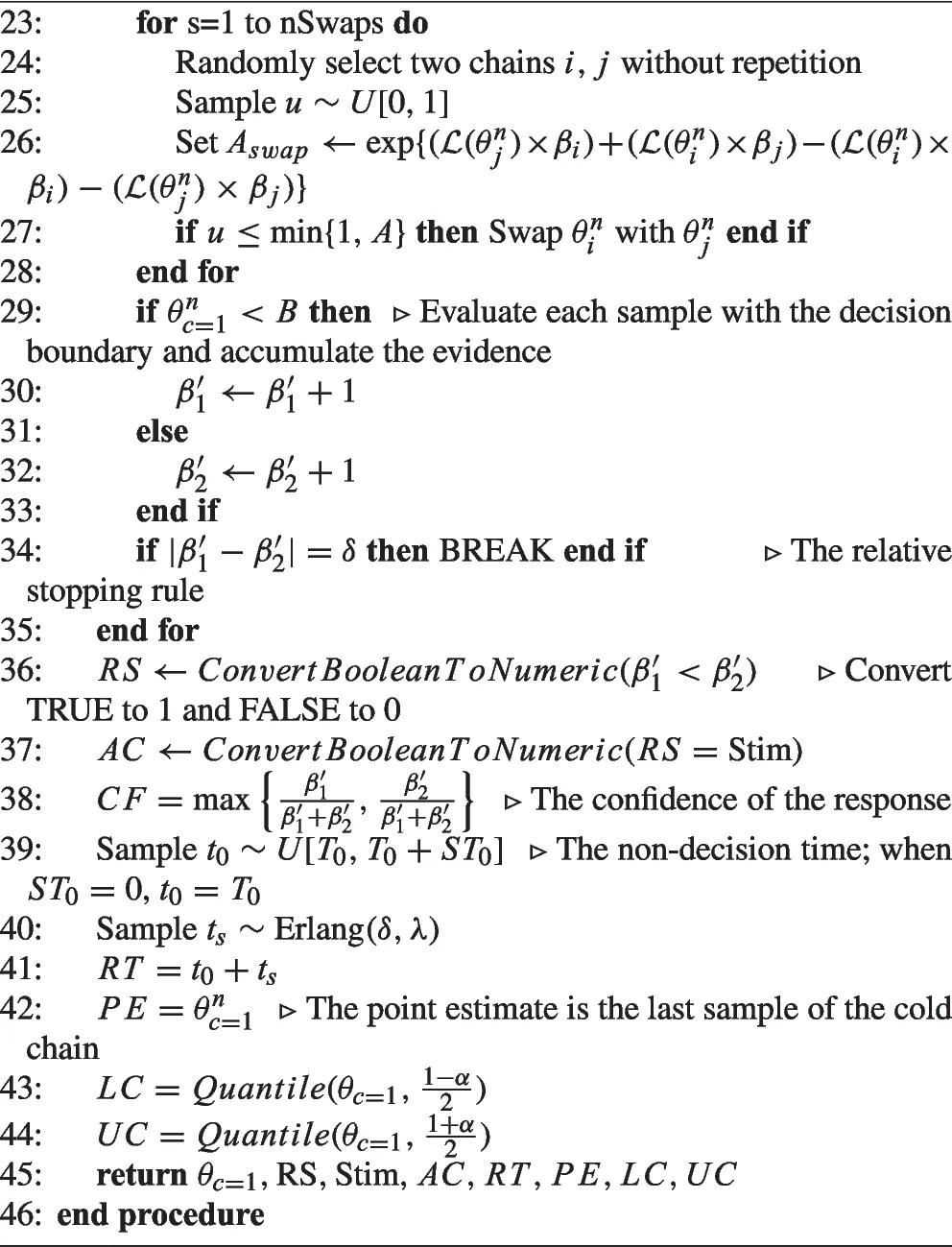
Autocorrelated Bayesian sampler with relative stopping rule for one trial (continued)

## Data Availability

Code used to produce the figures in this article is available at https://github.com/lucas-castillo/samplr-paper.

## Appendix A Implementation details

### Sampling algorithms

The sampling algorithms offered by the samplr package are Markov chain Monte Carlo algorithms (MCMC; Brooks, Gelman, Jones, & Meng, 2011). These work by initializing a Markov chain at a random point, then having the chain transition between points in a way that guarantees that, after sufficient iterations, the distribution of registered states in the chain will approximate the target distribution^1^. They can be understood as stemming from a base algorithm, Metropolis–Hastings (MH; Hastings, Hastings (1970)), to which qualitative modifications can be made (see Table 3). Below, we describe MH and show how other algorithms originate from modifying it.

#### Metropolis–Hastings

After the Markov chain, θ, is initialized at a random point $$\theta ^1$$, the Metropolis–Hastings algorithm proceeds iteratively as follows: in each iteration, it first proposes a random new location $\theta^{\prime }$ by updating the current state with the addition of random normal noise (*r*): $$\theta ' \leftarrow \theta ^{n-1} + r$$; $$r \sim N(0, \sigma ^2)$$, where *n* is the current iteration and σ, the standard deviation of the proposal distribution, is a free parameter (set by sigma_prop in the samplr functions). Then, it chooses whether to transition to $\theta^{\prime }$ or not stochastically, with probability $$\min \{1, exp\{\mathcal {L}(\theta ') - \mathcal {L}(\theta ^{n-1})\}\}$$, where $$\mathcal {L}(\cdot )$$ is the log probability density function of the target distribution (see Algorithm 1). Intuitively, the sampler is greedy, so that if $\theta^{\prime }$ is better than θ it will always transition to this new state; but also curious, so that when $\theta^{\prime }$ is worse the change is still possible.

**Table 3:** Sampling algorithms offered by the samplr package, and their qualitative features. *Note:* Algorithms marked with an asterisk do not have their own separate function, but can be used by setting the alpha parameter in the corresponding function to a value other than 0

Sampling algorithm	Function name	Multiple chains	Gradient-based proposals	Autocorrelated-proposals
MH	sampler_mh	No	No	No
MH_REC_^*^	sampler_mh	No	No	Yes
HMC	sampler_hmc	No	Yes	No
REC	sampler_rec	No	Yes	Yes
MC^3^	sampler_mc3	Yes	No	No
MC^3^_REC_^*^	sampler_mc3	Yes	No	Yes
MCHMC	sampler_mchmc	Yes	Yes	No
MCREC	sampler_mcrec	Yes	Yes	Yes

#### Multiple chains

One possible qualitative modification is running multiple chains that swap positions stochastically. Each chain *c* runs at different temperatures $\beta _c = 1 / (1 + \Delta _T \times (c-1))$, calculating the probability of a transition as $\min \{1, exp\{(\mathcal {L}(\theta ') - \mathcal {L}(\theta _c^{n-1})) \times \beta _c\}\}$, where $\Delta _T$ is the parameter (called delta_T in the samplr package) that controls how steeply the temperature increases. Then, in each iteration, after all chains have carried out both proposal and acceptance steps, a third swapping stage takes place: chains are randomly allocated to a pair, and for each pair, a position swap is proposed. These swaps are decided by comparing the joint likelihood of the current position-temperature pairs to what the joint likelihood would be if the chains swapped positions (lines 18–23 in Algorithm 2). If the swap is accepted, then the positions of the chains are exchanged. The number of chains to run is determined by a parameter *C* (called nChains in the package). Also, either all possible swaps or only one may be proposed at each iteration, which is determined by a third boolean parameter *SA* (called swap_all in the package). Although, when using this modification, the position of the sampler is defined by a N×C matrix θ where each row is an iteration and each column is one of the chains, only the positions of the cold chain ($\theta _{c=1}$) are returned.

#### Gradient-based proposals

Another modification to the Metropolis–Hastings proposal is to make proposals based on the gradient of the posterior distribution by simulating a physical system. Intuitively, algorithms using this modification will make proposals by simulating a frictionless particle in physical space, with the topology of the space being the negative log density (and thus its potential energy is proportional to that) and the kinetic momentum being drawn at random from a standard normal distribution at each iteration. Once the momentum has been initialized, the trajectory is simulated for a pre-set period of time, at which point the ending location is recorded as the new proposal.

In practice, the physical simulation needs to be carried out in discrete steps, by using a small stepsize ϵ and approximately computing the position at times $\epsilon , 2\epsilon , \dots , L\epsilon$ (a procedure for simulating the Hamiltonian equations while minimizing error is called the “leapfrog” method, which is shown in Algorithm 3). For this reason, the gradient-based samplers will use a measure of how long this trajectory will be simulated for as the product of $L \times \epsilon$ (these are called L and epsilon in the package).

Once a proposal has been made, the transition probability is determined by the joint density of the position-momentum pair (not just the density of the position), and so the proposal will be accepted with probability $$\min \{1, \frac{exp\{\mathcal {L}(\theta ') - \frac{1}{2}p'\cdot p'\}}{exp\{\mathcal {L}(\theta ^{n-1}) - \frac{1}{2}p\cdot p\}}\}$$. Notice that the last step of the leapfrog algorithm (line 8) is to negate the momentum $p^{\prime }$, which makes the Markov chain reversible.

If the sampler ran multiple chains, the swapping procedure in Algorithm 2, lines 17–22 would follow. Note that the joint density, not the density alone, should be used to calculate whether a swap should occur, and that the momentum variable for the i^th^ chain should be drawn from $p \sim N(0, 1 / \beta _i)$, not p∼N(0,1).

For more information on gradient-based proposals and the leapfrog method, see Neal (2011) and Betancourt (2018).

#### Autocorrelated proposals

The final possible modification implemented is to use autocorrelated proposals, as proposed by Horowitz (1991). A sampler using this approach uses one additional parameter, α, (called alpha in the samplr functions); $\alpha \in U[-1,1]$, with negative values leading to anticorrelated behavior and α=0 being uncorrelated behavior.

Algorithms with this modification and gradient-based proposals make a slight modification to how the momentum is drawn (ln. 15 in Algorithm 3), so that

1 $$\begin{aligned} p \leftarrow \alpha \times p^{n-1} + (1 - \alpha ^2)^{\frac{1}{2}} \times v; \hspace{5mm} v \sim N(0, 1) \end{aligned}$$

where $$p^{n-1}$$ is the momentum at the end of the last iteration ($p^{\prime }$). Because in the standard leapfrog algorithm the momentum is negated at the end, samplers with this modification will have to negate the momentum again (to undo the change of direction that changing the sign involves). In the first iteration of the sampler and after a chain has swapped position (in samplers with multiple chains) the momentum is not recycled but drawn from a standard normal distribution (as in HMC).

For sampling algorithms that do not use gradient-based proposals, this modification applies to how proposals are produced. While typically a proposed item is generated by adding normal unbiased noise *r* to $\theta ^{n-1}$ (line 4 in Algorithm 1), here a history of added noise is kept, and the noise added to $\theta ^{n-1}$ is a vector *r*, produced following Equation 1, substituting the following terms in this Equation: v∼N(0,1) with $v\sim N(0, \sigma )$ and *p* and $p^{n-1}$ with *r* and $r^{n-1}$; with $r^{n-1}$ being the noise that was added to $\theta ^{n-2}$ in the previous iteration. In the first iteration of the sampler and after a chain has swapped position (in samplers with multiple chains) *r* is drawn from $N(0, \sigma ^2)$.

### Cognitive models

The samplr package supports simulation operations for two cognitive models: the Bayesian sampler (BS; Zhu et al., 2020) and the autocorrelated Bayesian sampler (ABS; Zhu et al., 2024). In the sections below, we describe how BS generates probability judgments, and how ABS produces estimates, probability judgments, confidence intervals, choices, confidence judgments, and response times within a single trial.

#### Bayesian sampler

According to the BS, the mean and variance of a probability judgment can be analytically derived from the true probability *p* of the target event (i.e., the conjunctions and disjunctions of A and B in the Bayesian_Sampler function), the fixed sample size parameter *N* (the argument N) and the Beta prior β (the argument beta). To simulate a probability judgment, the model first draws a random value *X* from a Binomial distribution to represent the number of times the event occurs out of *N* samples. The final probability judgment is then computed according to the BS formula, incorporating *X*, *N*, and the symmetric beta prior β.

#### Autocorrelated Bayesian sampler

ABS implements two distinct stopping rules: for probability judgments, point estimates, and confidence intervals, it uses a fixed stopping rule, whereas for decisions involving two alternatives, it applies a relative stopping rule.

As shown in Algorithm 5, under the fixed stopping rule (specified via stopping_rule = ‘fixed’ in Zhu23ABS()), ABS draws a fixed number of samples δ (set by n_samples) using the MC^3^ sampler. Because the MC^3^ sampler requires a starting point $\theta ^1$ (called start_point), ABS will draw an initial value from a Gaussian distribution $N(\text {Stim}, \sigma _d^2)$ if the user does not provide one. Here, $\text {Stim}$ refers to the stimulus value (set via stimulus), and $\sigma _d$ is the standard deviation (given by distr_params).

The total decision time *RT* is computed as the sum of a non-decision time $t_0$ and a sampling time $t_s$. The non-decision time is drawn from a uniform distribution, while the sampling time follows an Erlang distribution. The point estimate *PE* is defined as the last sample in the sequence. The lower and upper bounds of a confidence interval at a given level α (given by conf_level) are computed from the quantiles of the sample sequence.

Under the relative stopping rule (specified via stopping_rule = ‘relative’ in Zhu23ABS()), ABS operates differently from the fixed stopping rule (see Algorithm 6). The process begins by generating a starting point $$\theta ^1$$ if the user does not provide one. However, in the relative stopping rule, the target distribution is defined as $$N(d'(\text {Stim}-\frac{1}{2}), \sigma _d^2)$$, where $d^{\prime }$ represents the discriminability of the two alternatives (called discrim in Zhu23ABS()). The stimulus $$\text {Stim}$$ must be either 0 or 1, indicating the two alternatives. Thus when $$\text {Stim} = 0$$, the target distribution becomes $$N(-\frac{d'}{2}, \sigma _d^2)$$; while when $$\text {Stim} = 1$$, the target distribution is $$N(\frac{d'}{2}, \sigma _d^2)$$.

ABS also incorporates a beta prior, $\text {Beta}(\beta _1, \beta _2)$ (defined by prior_on_resp in Zhu23ABS), which reflects the prior on responses before the evidence accumulation. Lines 5–9 of Algorithm 6 describe the mechanism of adaptive priors for repeated choices, which can be enabled or disabled using the prior_depend argument in Zhu23ABS. This mechanism allows the model to incorporate historical information into the prior. If there is no adaptive prior, the prior on responses for all the trials will be: $\beta _1' \leftarrow \beta _1,~\beta _2' \leftarrow \beta _2$. Lines 10–35 in Algorithm 6 mirror the steps of the MC^3^ algorithm, with the key difference that each sampled value will be compared against the decision boundary *B* (specified by dec_bdry). Evidence is accumulated based on the side of the boundary on which each sample falls. The relative difference between the accumulated evidence is then compared to the threshold δ (called delta), which determines when sampling stops.

Lines 37–40 in Algorithm 6 demonstrate how ABS generates responses, determines accuracy, and computes decision confidence. The remaining steps are identical to those in the fixed stopping rule implementation.

## Code Availability

The software here presented is available in the CRAN repository (https://cran.r-project.org/web/packages/samplr/), and its development version is available at https://github.com/lucas-castillo/samplr.
